# Sustainable triazine-derived quaternary ammonium salts as antimicrobial agents†

**DOI:** 10.1039/d1ra03455c

**Published:** 2021-08-19

**Authors:** Andrea Morandini, Emanuele Spadati, Benedetta Leonetti, Roberto Sole, Vanessa Gatto, Flavio Rizzolio, Valentina Beghetto

**Affiliations:** Università Ca’ Foscari di Venezia Via Torino 155 Venezia Mestre 30172 Italy beghetto@unive.it; Brenta S.r.l. – Nine Trees Group. Viale Milano, 26 36075 Montecchio Maggiore Vicenza Italy; Consorzio Interuniversitario per le Reattività Chimiche e Catalisi (CIRCC) Via C. Ulpiani 27 70126 Bari Italy; Crossing S.r.l. Viale della Repubblica 193/b Treviso 31100 Italy

## Abstract

The first examples of highly efficient antimicrobial triazine-derived bis imidazolium quaternary ammonium salts (TQAS) are reported. TQAS have been prepared with an easy, atom efficient, economically sustainable strategy and tested as antimicrobial agents, reaching MIC values below 10 mg L^−1^. Distinctively, TQAS have low MIC and low cytotoxicity.

## Abstract 2

The first examples of highly efficient antimicrobial triazine-derived bis imidazolium quaternary ammonium salts (TQAS) are reported.

## Graphical abstract

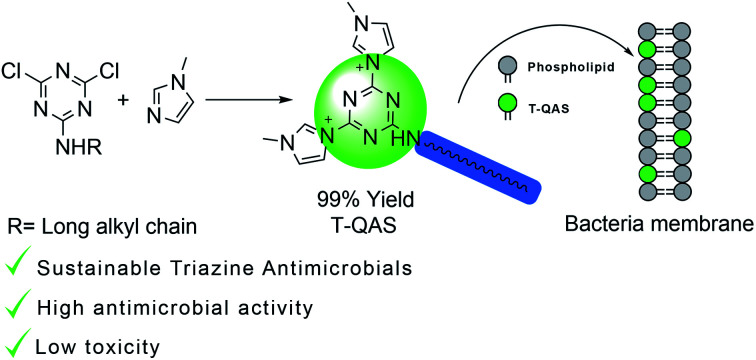

Infectious diseases remain one of the most important health problems in the world affecting millions of people.^1^ The new COVID-19 pandemic further increased the pressure for the development of new efficient antibacterial agents to prevent the spread of pathogens from the environment to human beings. Surface disinfection emerges as a winning strategy towards the mitigation of multi-drug resistance (MDR) phenomena, preventing the spread of pathogens. A wide variety of physical^2^ and chemical^3^ treatments have been developed to combat the spreading of bacterial infections.

Antibiotics have been massively employed to face global health emergencies,^4^ but abuse of antibiotics has generated multi drug resistant bacteria, hampering the efficiency of infection treatments.^5^ Thus the development of alternative antimicrobials is fundamental for human survival. Additionally, because of the COVID-19 pandemic, the use of surface disinfectants has emerged as a fundamental best practice for prevention.

Quaternary ammonium salts (QAS) are a well-known class of efficient antimicrobial agents with a broad spectrum of activity and long-lasting efficiency due to their persistence on treated surfaces.^1*a*,4*b*,6^ They are commonly present in domestic cleaning products,^6*g*^ food preservatives,^7^ health care products,^6*d*,8^ biomedical materials^9^ and surfactants,^10^ and are expected to reach a turnover of $8 billion by 2021.^11^

QAS generally contain four alkyl, aryl, or heterocyclic organic groups bonded to a nitrogen atom. According to the literature, the organic groups present on the nitrogen atom play a pivotal role in determining the antimicrobial action of QAS, requiring an hydrophobic segment compatible with the bilayer of the membrane and a positively charged quaternary nitrogen atom alike to the polar head of phospholipid acids.^9,12^ Thus, at least one of the organic substituents present on the nitrogen atom should be an alkyl chain and in fact, the activity of QAS may be tuned by changing the length of this alkyl chain.^13,14^ Additionally, the antimicrobial activity of QAS depends on the number of positive charges present, so that these compounds are divided into Mono, Bis (gemini) and tris-QAS (Fig. 1).^6*a*,*c*,8,9,14^

**Fig. 1:**
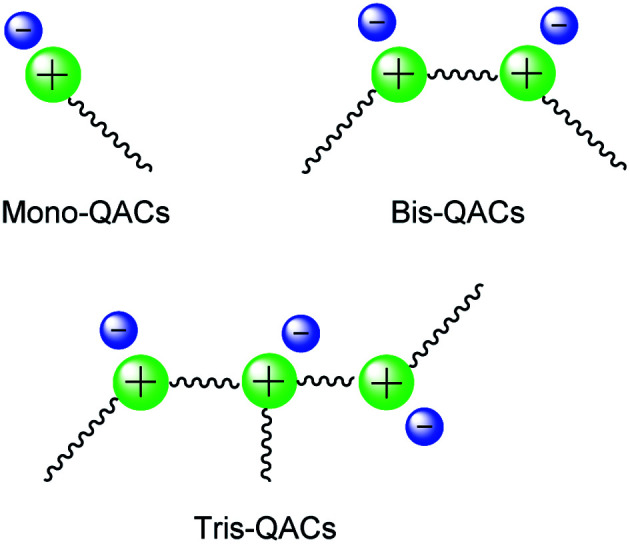
General QAS classification.

Bis-QAS are in general more efficient than mono QAS, while no significant improvement is often observed when tris-QAS are employed. Additionally, bis-QAS induce lower bacterial resistance phenomena compared to most commercially available mono-QAS.^6*c*–15^ Generally, QAS are synthesized by reaction of a tertiary amine with an alkyl, aryl, or heterocyclic halide at solvent reflux temperature. This standard procedure, despite its simplicity, in most cases, requires the use of toxic alkyl-bromides as reagents.

With all this in mind, herein we reported the synthesis of new triazine based bis-QAS and their efficacy as antimicrobial agents against several Gram-positive and Gram-negative bacterial species. To the best of our knowledge, this is the first example of the use of triazine quaternary ammonium salts as antimicrobials. The use of a triazine core allowed us to implement an innovative synthetic approach avoiding the use of alkyl halides and reducing solvent consumption based on a simple, easy to tune, atom efficient methodology.

Triazines are very reactive compounds, widely employed for the synthesis of a plethora of fine chemicals, dyes, herbicides, polymers.^16^ Since 1998, when Kunishima and Kaminsky developed the synthesis of the first triazine derived quaternary ammonium salt, 4-(4,6-dimethoxy-1,3,5-triazin-2-yl)-4-methyl-morpholinium chloride (DMTMM), triazines have found a key application as activators in dehydro-condensation reactions for the production of amides or esters (Fig. 2).^17^ Nevertheless, DMTMM is a very reactive compound, with low stability in solution decomposing within 48 h, to generate 2,4-dimethoxy-6-hydroxy-1,3,5-triazine (DMTOH) and *N*-methyl morpholinium chloride. Thus, this class of quaternary ammonium salts are inadequate to be used as antimicrobials and have never been tested for the scope.

**Fig. 2:**
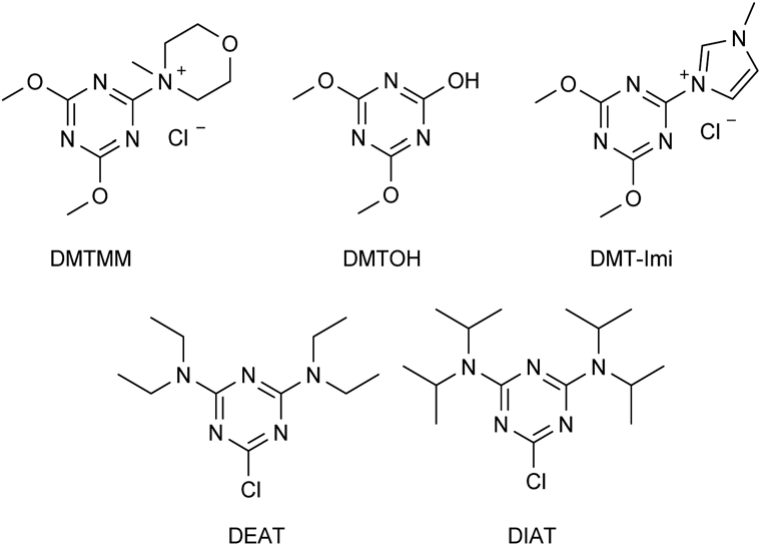
DMTMM, DMTOH, DMT-Imi, DEAT, DIAT.

It is no less true that according to the literature the stability and reactivity of 4-(4,6-dimethoxy-1,3,5-triazin-2-yl)-4-*tert*-amine chloride, prepared by reaction of 2-chloro-4,6-dialcoxy-1,3,5-triazine and a *tert*-amine, may be tuned by changing the *tert*-amine.^18^ When the *tert*-amine is *N*-methylimidazole, the corresponding 3-(4,6-dimethoxy-1,3,5-triazin-2-yl)-1-methyl-1*H*-imidazol-3-ium chloride (DMT-Imi, Fig. 2) is highly stable and is unable to promote condensation reactions.^18*a*^

With a radically different approach, this drawback became a positive feature with the aim to prepare stable triazine based quaternary ammonium salts to be used as antimicrobials. According to the general rule that antimicrobials should have a long alkyl chain within their structure and that bis-QAS are more efficient and give lower bacterial resistance phenomena, 3,3′-(6-(alkylamino)-1,3,5-triazine-2,4-diyl)bis(1-methyl-1*H*-imidazol-3-ium) quaternary ammonium salts were synthesized and tested as antimicrobials. Thus, two different synthetic approaches were devised for the synthesis of bis-TQAS (VI–X), as reported in Scheme 1.

**Scheme 1:**
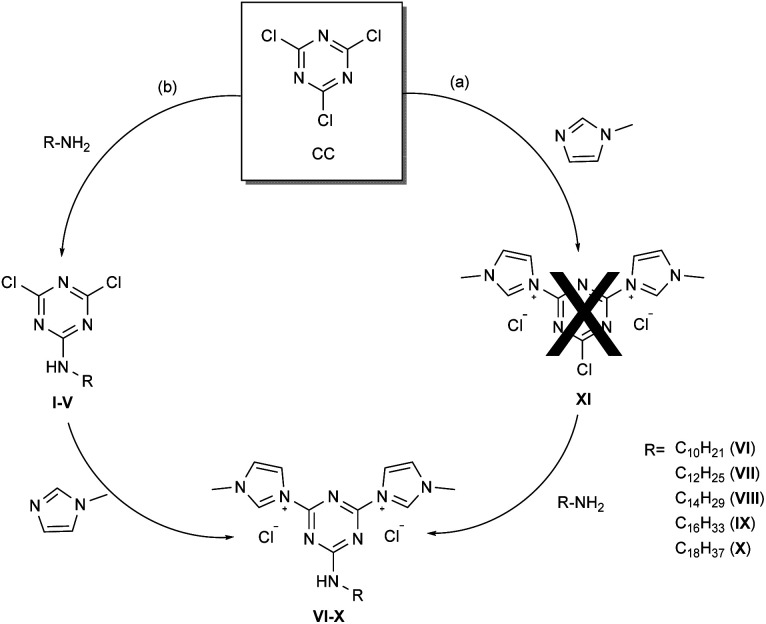
Alternative synthetic strategies for bis-TQAS.

The synthesis of bis-TQAS was tested by two different synthetic strategies employing the same reagents but in a different addition sequence (Scheme 1). In the first case, 2,4,6-trichloro-1,3,5-triazine (CC) was reacted, at room temperature, with methyl imidazole, in 1/2 molar ratio (Scheme 1a), but no 3,3′-(6-chloro-1,3,5-triazine-2,4-diyl)bis(1-methyl-1*H*-imidazol-3-ium) chloride (XI) was formed. Further experiments were carried out in different reaction conditions but without success.

A second synthetic strategy was verified, foreseeing first the synthesis of 4,6-dichloro-*N*-alkyl-1,3,5-triazin-2-amine (I–V) by reaction of CC with an alkyl amine, followed by the reaction of compounds I–V with *N*-methylimidazole (Scheme 1b). Commercially available alkyl amines of variable length (C10–C18) were used to introduce into final bis-TQAS a long hydrophobic carbon chain required to interact with the bilayer of the membrane (Schema 1b).^6*e*,19^ With this synthetic strategy five different 2,4-dichloro-6-alkylamino-1,3,5-triazines (I–V) were synthesized in yields between 50–70%, within 1.5 h (Scheme 1b). Addition of the primary amine was carried out at 0 °C to prevent the formation of di or tri-substituted products (Scheme 1, ESI†). The synthesis of bis-TQAS was carried out at room temperature, by simple addition of two equivalents of *N*-methylimidazole to compounds I–V, to give the desired bis-TQAS (VI–X) (Scheme 1b). No purification steps were required and all bis-TQAS were recovered from the reaction mixture, within few minutes, by simple filtration in high yields (≥90%).

Additionally, 2-chloro-4,6-dialkylamino-1,3,5-triazines could be prepared for the synthesis of corresponding mono-TQAS. Nevertheless, as reported in the literature, the presence of two alkylamino electron-donating groups strongly reduce the reactivity of the corresponding triazine, so much that, 2-chloro-4,6-(diethylamino)-1,3,5-triazine (DEAT) and 2-chloro-4,6-(diisopropylamino)-1,3,5-triazine (DIAT) are unable to react with a tertiary amine to give the corresponding triazine quaternary ammonium salt.^18*b*^ Thus, only 2,4-dichloro-6-alkylamino-1,3,5-triazines (I–V) were synthesized and further employed for the preparation of bis-TQAS.

The antimicrobial activity of all compounds was tested against Gram-positive *Staphylococcus aureus* (ATCC 25923), *Enterococcus faecalis* (CECT 795) and Gram-negative *Escherichia coli* (ATCC 25922)*, Pseudomonas aeruginosa* (CECT 111) strains. Minimum inhibitory concentration (MIC) was taken as lowest concentration completely inhibiting microbial growth and all experiments were carried out in biological and technical triplicate. Minimal inhibitory concentration, IC_50_, critical micelle concentration and Log *P*(*n*-octanol/water) of bis-TQAS are reported in Table 1.

**Table 1:** Minimal inhibitory concentration, IC_50_, critical micelle concentration and Log *P*(*n*-octanol/water) of TQAS

Compound	Log *P*	CMC [mM]	MIC [mg L^−1^]				IC_50_ [mg L^−1^]
			*S. a.*	*E. f.*	*E. c.*	*P. a.*	
VI	−0.77	—	250	125	250	500	13.3
VII	0.02	20.5	7.80	62.5	62.5	500	7.6
VIII	0.81	9.3	3.90	62.5	62.5	250	2.4
IX	1.60	2.2	125	62.5	250	>500	6.7
X	2.40	1.1	250	62.5	>500	>500	5.1

The activity of bis-TQAS towards *S. aureus* showed a parabolic trend for compound VI, IX and X, respectively with 10, 16, and 18 carbon atoms in the alkyl chain, while lowest MICs were measured with compounds VII and VIII (C12, C14). These data are well in line and even superior to most efficient antimicrobial bis-QAS recently reported in the literature, giving MIC values between 8 and 80 mg L^−1^.^20^

MIC against Gram-negative *E. coli* strains showed a similar trend to *S. aureus*, reaching best MIC (62 mg L^−1^) with bis-TQAS containing C12, C14 alkyl chains. Except for compound VI, all bis-TQAS were equally efficient towards *E. faecalis* with a MIC of 62 mg L^−1^ (Fig. 3 and 4). Moreover, VIII was moderately active also against *P. aeruginosa* (250 mg L^−1^). In fact, this Gram-negative strain is rather resistant to QAS and in most cases MIC values achieved are within the range of 40 to 1410 mg L^−1^, while only in few cases MIC values below 10 mg L^−1^ have been achieved, albeit with more complex structures and synthesis.^20^

**Fig. 3:**
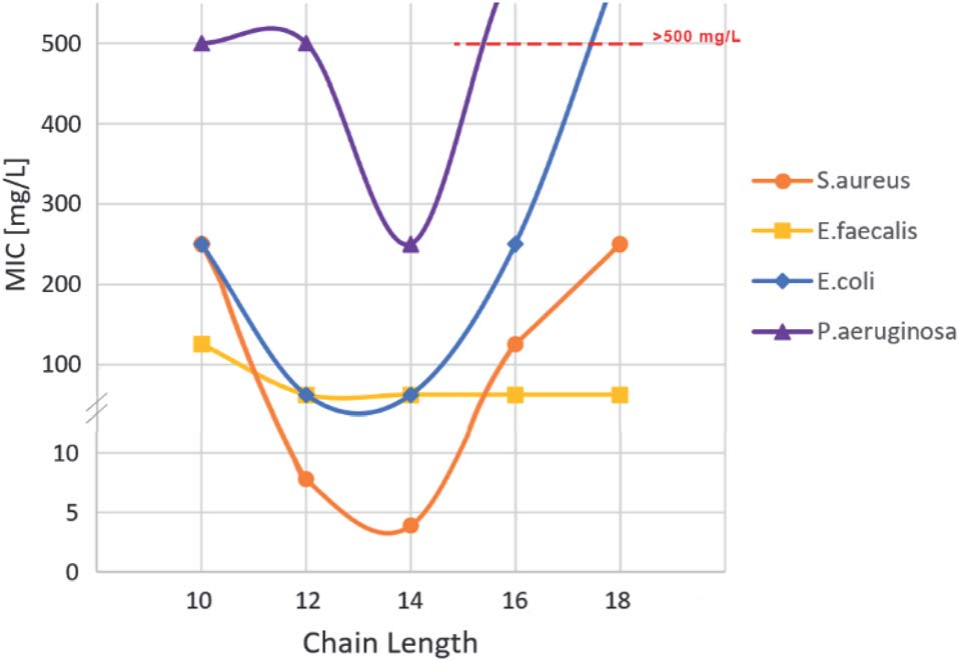
MIC values of different bis-TQAS against *S. aureus*, *E. faecalis*, *E. coli* and *P. aeruginosa*.

Cytotoxicity of bis-TQAS was tested against eukaryotic fibroblasts MRC-5 as half maximal inhibitory concentration IC_50_ values. IC_50_ for compounds VII, IX and X were comparable and respectively of 7.6, 6.7 and 5.1 mg L^−1^, while VIII, showed slightly higher cytotoxicity (2.4 mg L^−1^), but significantly lower than commercially available QAS such as for example benzalkonium chloride (0.1 μg mL^−1^),^20*b*^ indicating a very low cytotoxicity of bis-TQAS tested. Compound VI (C10), possessing a modest antimicrobial activity, showed even lower IC_50_ (13.3 mg L^−1^). Interestingly, MIC and IC_50_ are generally correlated to the alkyl chain length^19*b*^ while no significant variations were observed with bis-TQAS. Further studies are ongoing to investigate this behaviour, nevertheless it should be considered that correlation of biological effect and specific chain length may depend on several physicochemical parameters such as hydrophobicity, adsorption, solubility, and transport in the medium so that deviations from general structure–activity dependence have been reported for several QAS.^6*c*,21^

**Fig. 4:**
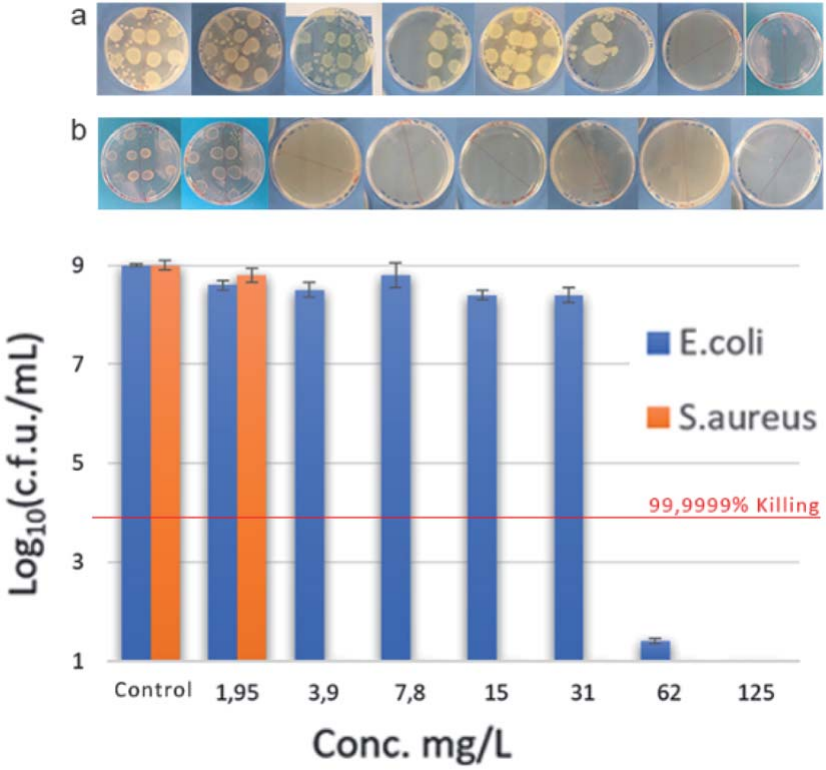
Antimicrobial performance of compound VIII at various concentrations against *S. aureus* (orange) and *E. coli* (blue).

To gain dipper insight into bis-TQAS activity, Log *P* and critical micelle concentration (CMC) of different bis-TQAS were calculated to determine possible correlations between hydrophobicity of the compounds and their antimicrobial activity. In fact, according to the literature^21,22^ the antimicrobial activity of QAS should depend not only on the length of the alkyl chain but also on CMC and hydrophobicity/lipophilicity.

MIC *versus* LogP showed a minimum for compounds VII and VIII (Fig. 5), confirming that, as for QAS, too long or too short alkyl chains adversely affect the antimicrobial activity of bis-TQAS, reaching highest MICs with bis-TQAS bearing twelve or fourteen carbon atoms. As far as CMC data are concerned, an elongation of the alkyl chain produced a progressive decrease of CMC values, ranging from 20.5 mM to 1.1 mM, respectively for compounds VI and X (Fig. 6).

**Fig. 5:**
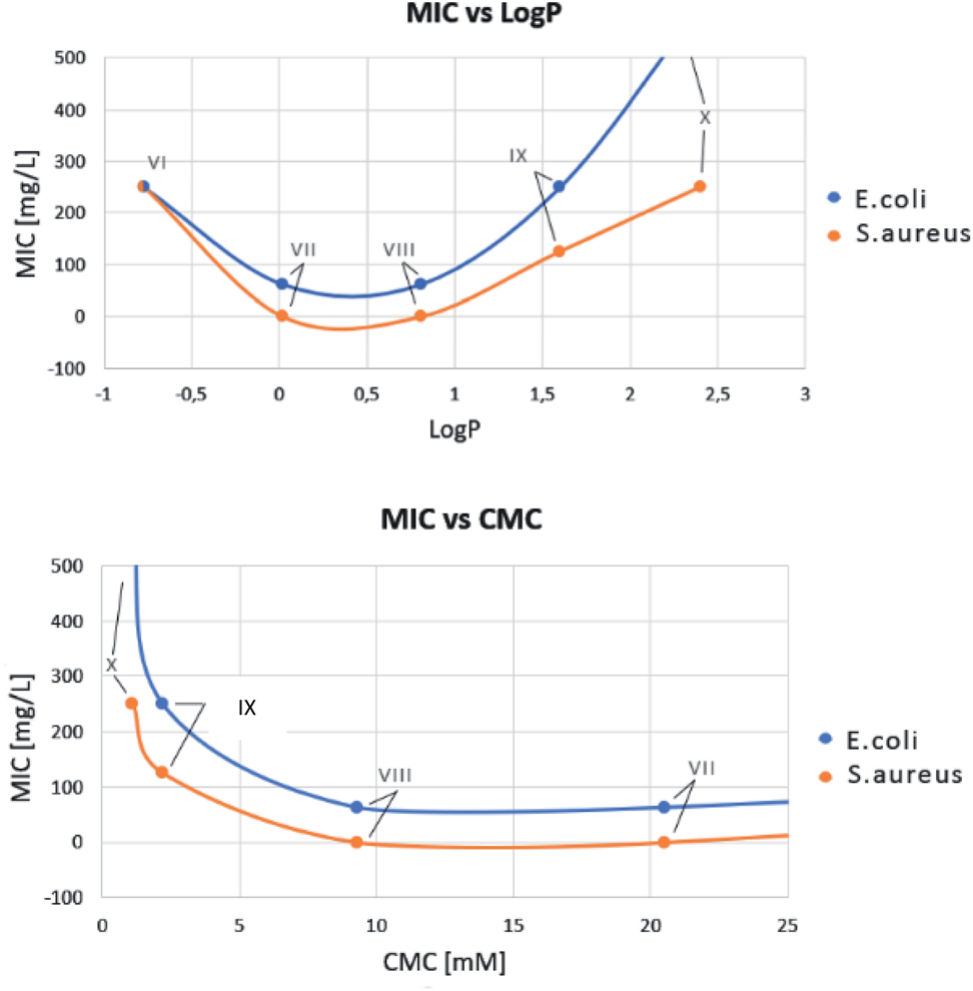
Bis-TQAS dependence of Log *P* (water/*n*-octanol) and CMC *versus* MIC, against *S. aureus* and *E. coli*.

**Fig. 6:**
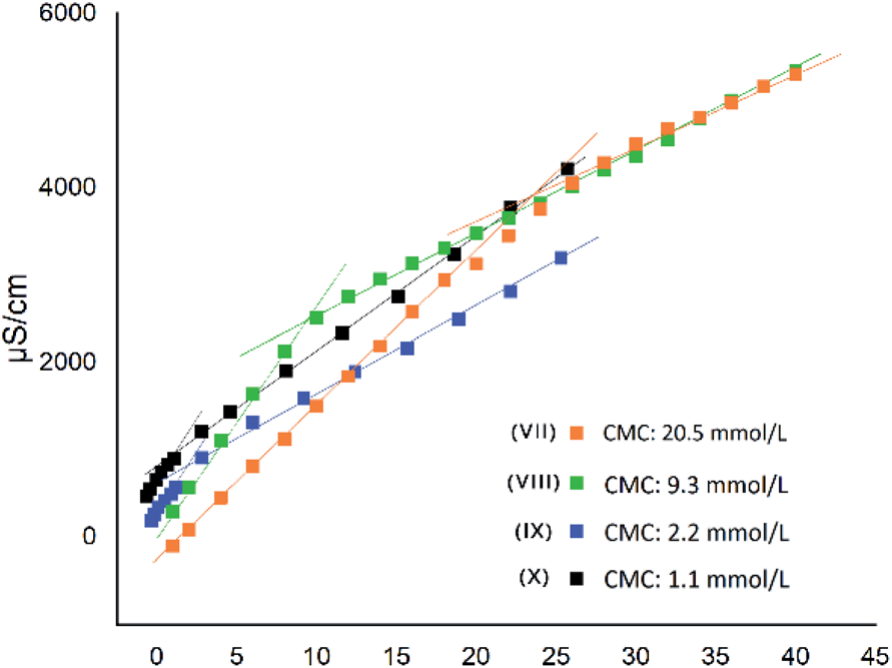
Conductivity measures of bis-TQAS, change in curve slope corresponds to CMC value.

For compounds, VII–VIII a decrease in CMC promoted an increase in MIC, due to improved adsorption of the hydrophobic chain by the microorganism membrane. Further CMC decrease observed with bis-TQAS IX and X (2.2 mM and 1.1 mM, Fig. 6), was associated with a decrease of the MIC values (Fig. 5). This peculiar behaviour, observed with other QAS,^21^ may be attributed to a reduced diffusion of higher molecular weight compounds and consequently lower CMC values were not associated with an improvement in antimicrobial activity.

## Conclusions

In conclusion, in this study, a family of stable bis-TQAS were synthesized starting from 2,4-dichloro-6-alkylamine-1,3,5-triazine, in the presence of methyl imidazole with an easy and sustainable methodology. The process was readily scaled up to over 500 g scale. The compounds have been obtained in very high yields (over 90%) in short reaction times (1.5 hours), without the use of alkyl halides. A set of different bis-TQAS was prepared bearing C10–C18 alkyl chains to study the correlation between the length of the alkyl chain and their antimicrobial activity against Gram-positive and Gram-negative bacterial strains.

The bacterial inhibition effects were found to be remarkable when bis-TQAS VII (C12) and VIII (C14) are in the range between 3.9 to 7.8 mg L^−1^ for *S. aureus*, and 62 mg L^−1^ for both *E. coli* and *E. faecalis*. A further benefit of these compounds is their low cytotoxicity compared to many commercially available QAS. This article therefore opens an innovative perspective to produce a new class of antimicrobial QAS having low toxicity.

## Conflicts of interest

There are no conflicts to declare.

## Supplementary Material

RA-011-D1RA03455C-s001
