# Supplementary material for: Sustainable triazine-derived quaternary ammonium salts as antimicrobial agents
Source: RSC Adv. 2021 Aug 19;11(45):28092–6. doi: 10.1039/d1ra03455c (PMC9038131; doi:10.1039/d1ra03455c)

Electronic Supplementary Material (ESI) for RSC Advances.
This journal is © The Royal Society of Chemistry 2021

Supporting Information

Sustainable Triazine derived quaternary ammonium salts as antimicrobial agents

Andrea Morandini, [a] Emanuele Spadati,[a] Benedetta Leonetti,[b] Roberto Sole,[a] Vanessa Gatto,[c] Flavio Rizzolio[a] and Valentina Beghetto*[a,c]

Table of Contents

I. General Information

II. Synthesis of Compounds

III. Biological Assays

IV. Determination of CMC and LogP

V. ^{1}H and ^{13}C NMR Spectra

I. GENERAL INFORMATION

All of the solvents and reagents were purchased from Sigma-Aldrich, no further purification was performed. All reactions were carried out under ambient atmosphere. The NMR spectra were recorded using a Bruker Advance 300 spectrometer operating at a frequency of 300.13 MHz for the proton spectrum and 75.4 MHz for the carbon spectrum, chemical shifts were reported on a δ-scale (ppm) downfield from TMS. The deuterate solvents used to perform the NMR spectra were; $CDCl_3$, D_2O, $(CD_3)_2CO$ and $(CD_3)_2SO$, with an internal reference of 7.26 ppm, 4.79 ppm, 2.05 ppm and 2.50 ppm for ^{1}H NMR and 77.16 ppm, 29.84 ppm, 39.52 ppm for ^{13}C NMR respectively. High-Resolution mass spectrometry data was obtained with an Agilent technologies InfinityLab HPLC-ESI/MS and GC-MS Agilent technologies 7820A GC system with A.T. 5977B MSD. The melting points were performed with the instrument Buchi 235.

II. *SYNTHESIS OF COMPOUNDS*

General procedure for preparation of 4,6-dichloro-*N*-alkyl-1,3,5-triazin-2-amine (I-V)

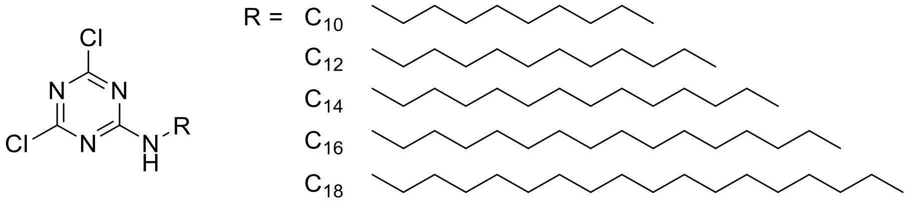

In a two-neck flask equipped with ice bath and magnetic stirring, 10 mmol of cyanuric chloride, 20 mmol of sodium bicarbonate and 50 mL acetone was introduced. Then, keeping the system under agitation, 10 mmol of alkyl-1-amine, was introduced drop by drop.

Then, the ice bath was removed and the mixture was kept under stirring for about one and a half hours. At the end of the reaction excess of $NaHCO_3$ and NaCl subproduct was removed by filtration with paper, and the solvent removed by rotary evaporator. The resulting solid is then purified by reprecipitation with hexane.

The product obtained has been characterized by ^{1}H NMR, ^{13}C NMR, FT-IR, GC-MS and melting point.

1) 4,6-dichloro-*N*-decyl-1,3,5-triazin-2-amine (I)

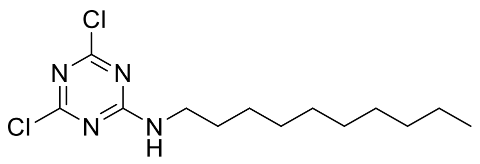

4,6-dichloro-*N*-decyl-1,3,5-triazin-2-amine
Chemical Formula: $C_{13}H_{22}Cl_2N_4$
Molecular Weight: 305,25

(I)

Yeld: 51%, **m.p**.: 61°C, **^{1}H NMR** (300 MHz, $CDCl_3$) δ (ppm): 6.05 (1H, s), 3.45 (2H, m), 1.60 (2H, q), 1.23 (14H, m,), 0.85 (3H, m), **^{13}C NMR** (75 MHz, CDCl3) δ (ppm): 171.13, 169.90, 165.96, 41.72, 32.01, 29.62, 26.77, 22.81, 14.25, **FT-IR** (KBr, cm-1): 3264 (vNH), 2921-2851 (vCH), 1620 (vC=N), 851 (vCCl), **GC-MS** (m/z): 304.100 $[M]^+$, 269.140 $[M - Cl]^+$, 233.050 $[M - 2Cl]^+$, 176.940 $[M - C9H19]^+$

2) 4,6-dichloro-*N*-dodecyl-1,3,5-triazin-2-amine (II)

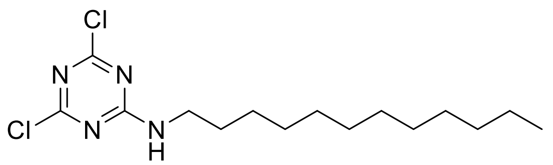

4,6-dichloro-*N*-dodecyl-1,3,5-triazin-2-amine
Chemical Formula: $C_{15}H_{26}Cl_2N_4$
Molecular Weight: 333,30

(II)

Yeld: 69%, **m.p**.: 70°C, **^{1}H NMR** (300 MHz, CDCl3) δ (ppm): 6.42 (1H, s), 3.44 (2H, q), 1.59 (2H, m), 1.23 (18H, s), 0.85 (3H, t), **^{13}C NMR** (75 MHz, CDCl3) δ (ppm): 171.07, 169.80, 165.92, 41.70, 32.02, 29.73, 26.77, 22.80, 14.22, **FT-IR**: (KBr, cm-1): 3266 (vNH), 2919-2850 (vCH), 1630 (vC=N), 848 (vCCl), **GC-MS** (m/z): 332.170 $[M]^+$, 297.210 $[M - Cl]^+$, 261.14 $[M - 2Cl]^+$, 177.010 $[M – C11H23]^+$

3) 4,6-dichloro-*N*-tetradecyl -1,3,5-triazin-2-amine (III)

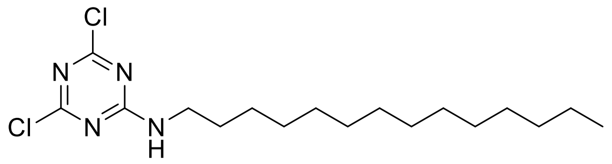

4,6-dichloro-*N*-tetradecyl-1,3,5-triazin-2-amine
Chemical Formula: $C_{17}H_{30}Cl_2N_4$
Molecular Weight: 361,36

(III)

Yeld: 52%, **m.p**.: 75°C (decomposition), **^{1}H NMR** (300 MHz, CDCl3) δ (ppm): 5.86 (1H, s), 3.48 (2H, q), 1.60 (2H, m), 1.26 (22H, s), 0.88 (3H, t), **^{13}C NMR** (75 MHz, CDCl3) δ (ppm): 171.16, 169.87, 165.98, 41.70, 32.02, 29.77, 26.77, 22.80, 15.06, **FT-IR:** (KBr, cm-1): 3267 (vNH), 2919-2850 (vCH), 1630 (vC=N), 848 (vCCl), **GC-MS** (m/z): 360.210 $[M]^+$, 325.260 $[M - Cl]^+$, 289.210 $[M - 2Cl]^+$, 177.020 $[M – C11H23]^+$

4) 4,6-dichloro-*N*-hexadecyl-1,3,5-triazin-2-amine (IV)

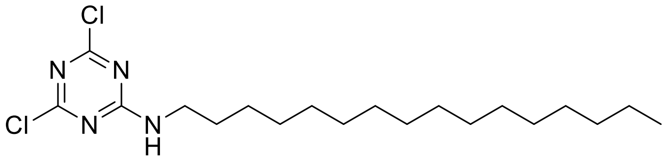

4,6-dichloro-*N*-hexadecyl-1,3,5-triazin-2-amine
Chemical Formula: $C_{19}H_{34}Cl_2N_4$
Molecular Weight: 389,41

(IV)

Yeld: 71%, **m.p.**: 69°C, **^{1}H NMR** (300 MHz, CDCl3) δ (ppm): 6.15 (1H, s), 3.47 (2H, q), 1.60 (2H, m), 1.24 (26H, s), 0.87 (3H, t), **^{13}C NMR** (75 MHz, CDCl3) δ (ppm): 171.18, 169.91, 165.98, 41.70, 32.02, 29.74, 26.77, 22.80, 14.24, **FT-IR**: (KBr, cm-1): 3266 (νNH), 2919-2850 (νCH), 1629 (νC=N), 848 (νCCl), **GC-MS** (m/z): 388.220 $[M]^+$, 353.260 $[M - Cl]^+$, 317.23 $[M - 2Cl]^+$, 176.960 $[M – C11H23]^+$

5) 4,6-dichloro-*N*-octadecyl -1,3,5-triazin-2-amine (V)

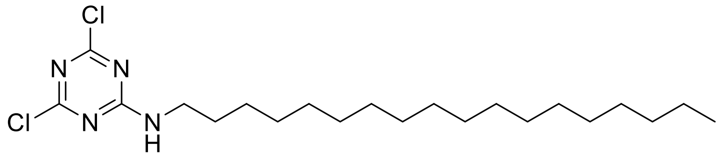

4,6-dichloro-*N*-octadecyl-1,3,5-triazin-2-amine
Chemical Formula: $C_{21}H_{38}Cl_2N_4$
Molecular Weight: 417,46

(V)

Yeld: 50%, **m.p.**: 80°C, **^{1}H NMR** (300 MHz, CDCl3) δ (ppm): 5.88 (1H, s), 3.46 (2H, q), 1.60 (2H, m), 1.25 (30H, s), 0.87 (3H, t), **^{13}C NMR** (75 MHz, CDCl3) δ (ppm): 171.19, 169.93, 166.01, 41.70, 32.02, 29.72, 26.77, 22.80, 14.30, **FT-IR**: (KBr, cm-1): 3266 (νNH), 2918-2849 (νCH), 1630 (νC=N), 847 (νCCl), **GC-MS** (m/z): 416.280 $[M]^+$, 381.330 $[M - Cl]^+$, 345.28 $[M - 2Cl]^+$, 176.980 $[M – C11H23]^+$

General procedure for preparation of 3,3'-(6-(alkylamino)-1,3,5-triazine-2,4-diyl)bis(1-methyl-1*H*-imidazol-3-ium) chloride (VI-X)

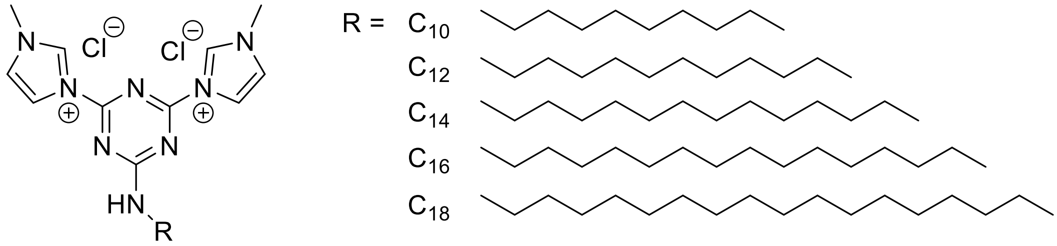

In a 100 ml one-neck flask with magnetic stirring, 3 mmol of 4,6-dichloro-*N*-alkyl-1,3,5-triazin-2-amine (**I**,**II**,**III**,**IV**,**V**) and 25 mL of acetone were added. Then 15 mmol of methylimidazole (MImi) was added drop by drop. The reaction was carried out for one and a half hours until a precipitate is formed. The solid is recovered by filtration on Gooch funnel and rinsed with a small amount of cold acetone, then the solids were drayed into a vacuum dryer. The solids obtained has been characterized by ^{1}H NMR, ^{13}C NMR, FT-IR and melting point.

6) 3,3'-(6-(decylamino)-1,3,5-triazine-2,4-diyl)bis(1-methyl-1*H*-imidazol-3-ium) chloride (VI)

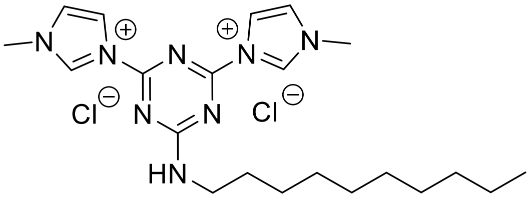

3,3'-(6-(decylamino)-1,3,5-triazine-2,4-diyl)bis(1-methyl-1*H*-imidazol-3-ium) chloride
Chemical Formula: $C_{21}H_{34}Cl_2N_8$
Exact Mass: 468,23

(VI)

Yeld: 86%, **m.p.:** 196°C (decomposition), **^{1}H NMR** (300 MHz, D_2O) δ (ppm): 8.40 (2H, d), 8.34 (2H, d), 7.69 (2H, dd), 4.09 (6H, s), 3.63 (2H, m), 1.70 (2H, m), 1.23 (14H, m), 0.83 (3H, m), **^{13}C NMR** (75 MHz, $CDCl_3$) δ (ppm): 164.51, 158.76, 158.07, 123.24, 117.50, 117.37, 35.09, 29.54, 26.98, 26.29, 24.11, 20.38, 11.75, **FT-IR** (KBr, cm^{-1}): 3042 (ν_{NH}), 2924-2852 (ν_{CH}), 1626 ($\nu_{C=N}$), 1343 (ν_{C-N}), 801 (ν_{CH}), **ESI-MS** (m/z): 199.1$[M]^{2+}$.

7) 3,3'-(6-(dodecylamino)-1,3,5-triazine-2,4-diyl)bis(1-methyl-1*H*-imidazol-3-ium) chloride (VII)

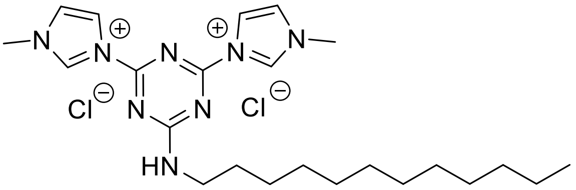

3,3'-(6-(dodecylamino)-1,3,5-triazine-2,4-diyl)bis(1-methyl-1*H*-imidazol-3-ium) chloride
Chemical Formula: $C_{23}H_{38}Cl_2N_8$
Exact Mass: 496,26

(VII)

Yeld: 97%, **m.p.:** 196°C (decomposition), **^{1}H NMR** (300 MHz, DMSO) δ (ppm): 10.68 (1H, s), 10.61 (1H, s), 9.55 (1H, t), 8.73 (1H, s), 8.43 (1H, s), 8.05 (1H, s), 8.02 (1H, s), 4.04 (6H, s), 3.53 (2H, q), 1.61 (2H, m), 1.24 (18H, s), 0.85 (3H, t), **^{13}C NMR** (75 MHz, $CDCl_3$) δ (ppm): 165.59, 159.84, 159.37, 137.81, 125.43, 125.31, 118.93, 118.51, 36.74, 31.25, 28.97, 28.75, 28.66, 26.25, 22.04, 13.91, **FT-IR** (KBr, cm^{-1}): 3040 (ν_{NH}), 2921-2851 (ν_{CH}), 1626 ($\nu_{C=N}$), 1341 (ν_{C-N}), 801 (ν_{CH}), **ESI-MS** (m/z): 213.2$[M]^{2+}$.

8) 3,3'-(6-(tetradecylamino)-1,3,5-triazine-2,4-diyl)bis(1-methyl-1*H*-imidazol-3-ium) chloride (VIII)

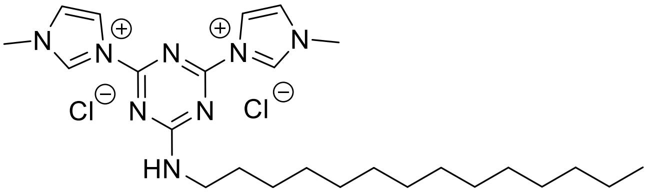

3,3'-(6-(tetradecylamino)-1,3,5-triazine-2,4-diyl)bis(1-methyl-1*H*-imidazol-3-ium) chloride
Chemical Formula: $C_{25}H_{42}Cl_2N_8$
Exact Mass: 524,29

(VIII)

Yeld: 97%, **m.p.:** 177°C (decomposition), **^{1}H NMR** (300 MHz, D_2O) δ (ppm): 8.37 (2H, d), 8.32 (2H, d), 7.72 (2H, dd), 7.69 (2H, dd), 4.09 (6H, s), 3.61 (2H, m), 1.69 (2H, m), 1.23 (22H, m), 0.85

(3H, m), **^{13}C NMR** (101 MHz, D_2O) δ (ppm): 168.53, 162.87, 162.12, 127.86, 121.61, 121.40, 44.20, 39.66, 39.51, 34.41, 32.36, 32.34, 32.25, 31.97, 31.92, 31.08, 29.40, 25.11, 16.39., **FT-IR** (KBr, cm^{-1}): 3035 ($ν_{NH}$), 2920-2850 ($ν_{CH}$), 1624 ($ν_{C=N}$), 1340 ($ν_{C-N}$), 801 ($ν_{CH}$), **ESI-MS** (m/z): 227.2[M] $^{2+}$.

9) 3,3'-(6-(hexadecylamino)-1,3,5-triazine-2,4-diyl)bis(1-methyl-1*H*-imidazol-3-ium) chloride (IX)

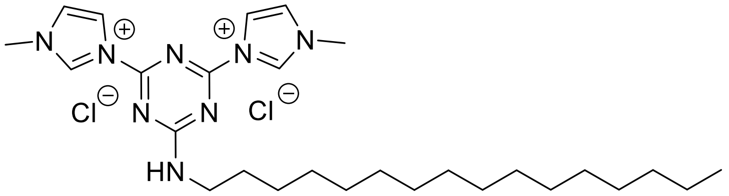

3,3'-(6-(hexadecylamino)-1,3,5-triazine-2,4-diyl)bis(1-methyl-1*H*-imidazol-3-ium) chloride
Chemical Formula: $C_{27}H_{46}Cl_2N_8$
Exact Mass: 552,32

(IX)

Yeld: 82%, **m.p.:** 200°C (decomposition), **^{1}H NMR** (300 MHz, D_2O) δ (ppm): 10.92 (1H, s), 10.89 (1H, s), 9.66 (1H, t), 8.99 (1H, s), 8.79 (1H, s), 8.09 (1H, s), 8.06 (1H, s), 4.05 (6H, s), 3.54 (2H, q), 1.59 (2H, m), 1.22 (26H, s), 0.84 (3H, t), **^{13}C NMR** (101 MHz, CDCl3) δ (ppm): 168.34, 162.71, 161.95, 127.25, 122.00, 121.57, 44.45, 39.83, 39.62, 37.99, 34.80, 32.99, 32.42, 31.23, 29.82, 25.38, 16.49., **FT-IR** (KBr, cm^{-1}): 3035 ($ν_{NH}$), 2918-2850 ($ν_{CH}$), 1628 ($ν_{C=N}$), 1349 ($ν_{C-N}$), 801 ($ν_{CH}$), **ESI-MS** (m/z): 241.3[M] $^{2+}$.

10) 3,3'-(6-(octadecylamino)-1,3,5-triazine-2,4-diyl)bis(1-methyl-1*H*-imidazol-3-ium) chloride (X)

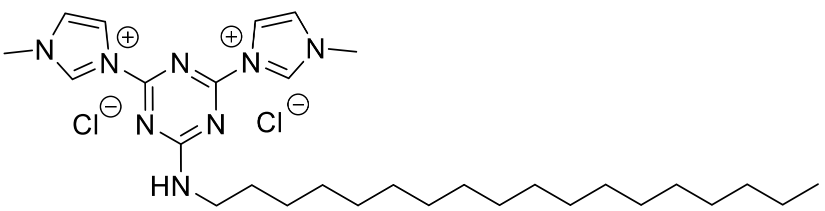

3,3'-(6-(octadecylamino)-1,3,5-triazine-2,4-diyl)bis(1-methyl-1*H*-imidazol-3-ium) chloride
Chemical Formula: $C_{29}H_{50}Cl_2N_8$
Exact Mass: 580,35

(X)

Yeld: 99%, **m.p.:** 220°C, **^{1}H NMR** (300 MHz, DMSO) δ (ppm): 10.88 (1H, s), 10.84 (1H, s), 9.63 (1H, t), 8.05 (1H, s), 7.96 (1H, s), 7.56 (1H, s), 7.47 (1H, s), 4.05 (6H, s), 3.52 (2H, q), 1.60 (2H, m), 1.22 (30H, s), 0.84 (3H, t), **^{13}C NMR** (101 MHz, $CDCl_3$) δ (ppm): 168.32, 162.67, 161.91, 127.22, 121.50, 121.09, 44.37, 39.83, 39.63, 34.67, 32.81, 32.23, 31.16, 29.75, 25.29, 16.50**FT-IR** (KBr, cm^{-1}): 3089 ($ν_{NH}$), 2918-2850 ($ν_{CH}$), 1631 ($ν_{C=N}$), 1353 ($ν_{C-N}$), 801 ($ν_{CH}$)., **ESI-MS** (m/z): 255.3[M] $^{2+}$.

III. BIOLOGICAL ASSAYS

All the culture media and pathogenic strains were purchased from Sigma Aldrich. The bacterial and fungal strains were stored at -20°C in glycerol stocks before the use.

Antimicrobial test

The screening of antimicrobial activity of these new molecules was conducted using a 96-well microplate against *Staphylococcus aureus* ATCC 25923, *Escherichia coli* ATCC 25922, *Enterococcus faecalis* CECT 795, *Pseudomonas aeruginosa* CECT 111. Specifically, we used as culture media Nutrient Broth for *S. aureus*, Tryptic Soy Broth for *P. aeruginosa* and *E. coli*, Brain Heart Infusion for *E. faecalis*. In particular, we made 1:2 serial dilutions from 0.5 to 0,0625 mg/ml of each synthesized compound. After, we inoculated 10^5 CFU/ml of overnight culture of each strain of in a final volume of 0.180 ml of liquid culture medium for each well. The microplate was incubated overnight under stirring (150 rpm) in a thermoshaker at 37°C for bacterial strains. Serial dilutions from 10^{-6} to 10^{-1} of each well were spotted on Petri dishes using spot plating technique.* The Petri dishes were incubated overnight at 37°C. The Minimum inhibitory concentration (MIC) was taken as the lowest concentration that completely inhibits microbial growth.

* J. M. Whitmire, D. S. Merrell *Humana Press* **2012**, pp. 17-27.

Cell viability assay (IC_{50})

MRC-5 fibroblast cells (from ATCC) were maintained at 37 °C in a humidified atmosphere containing 5% CO_2 according to the supplier. Cells (8×10^2) were plated in 96-well culture plates. The day after seeding, vehicle or compounds were added at different concentrations to the medium. Compounds were added to the cell culture at a concentration ranging from 1000 µg/ml to 0.32 µg/ml. Cell viability was measured after 96 h according to the supplier (CellTiter-Glo® luminescence assay, Promega G7571) with a Tecan M1000 PRO instrument. IC_{50} values were calculated from logistical dose response curves and performed in triplicates (GraphPad Prism).

Table S1. IC_{50} values of compounds **VI-X**.

Compound	IC_{50} (mg/L)	Std Dev
**VI**	13.33	3.92
**VII**	7.62	1.09
**VIII**	2.45	0.19
**IX**	6.75	0.68
**X**	5.14	1.58

Statistical analysis

All of the presented data are given as the mean value with the standard deviation (SD) on the basis of at least three independent measurements. Multifactor analysis of variance (ANOVA) was performed to evaluate the statistical variability. Statistical significance was set at the level of $p < 0.05$.

IV. DETERMINATION OF CMC AND LogP

Determination of critical micelle concentration

A series of 50 mL variable concentration solutions (40, 38, 36, 34, 32, 30, 28, 26, 24, 22, 20, 18, 16, 14, 12, 10, 8, 6, 4, 2, 1, 0.8, 0.6, 0.4, 0.2, 0.1 mM) of **VI-X** in milliQ water were prepared. Using

a XS instruments COND 80+ conductometer calibrated with standards KCl solutions. The conductivity value of each solution was recorded at 25 °C. The conductivity values were then plotted on a μS vs mM graph. The CMC value is marked by the change in slope of the straight line. The intersection of the two lines with different slopes whose values were obtained on Excel calculation software indicates the CMC.

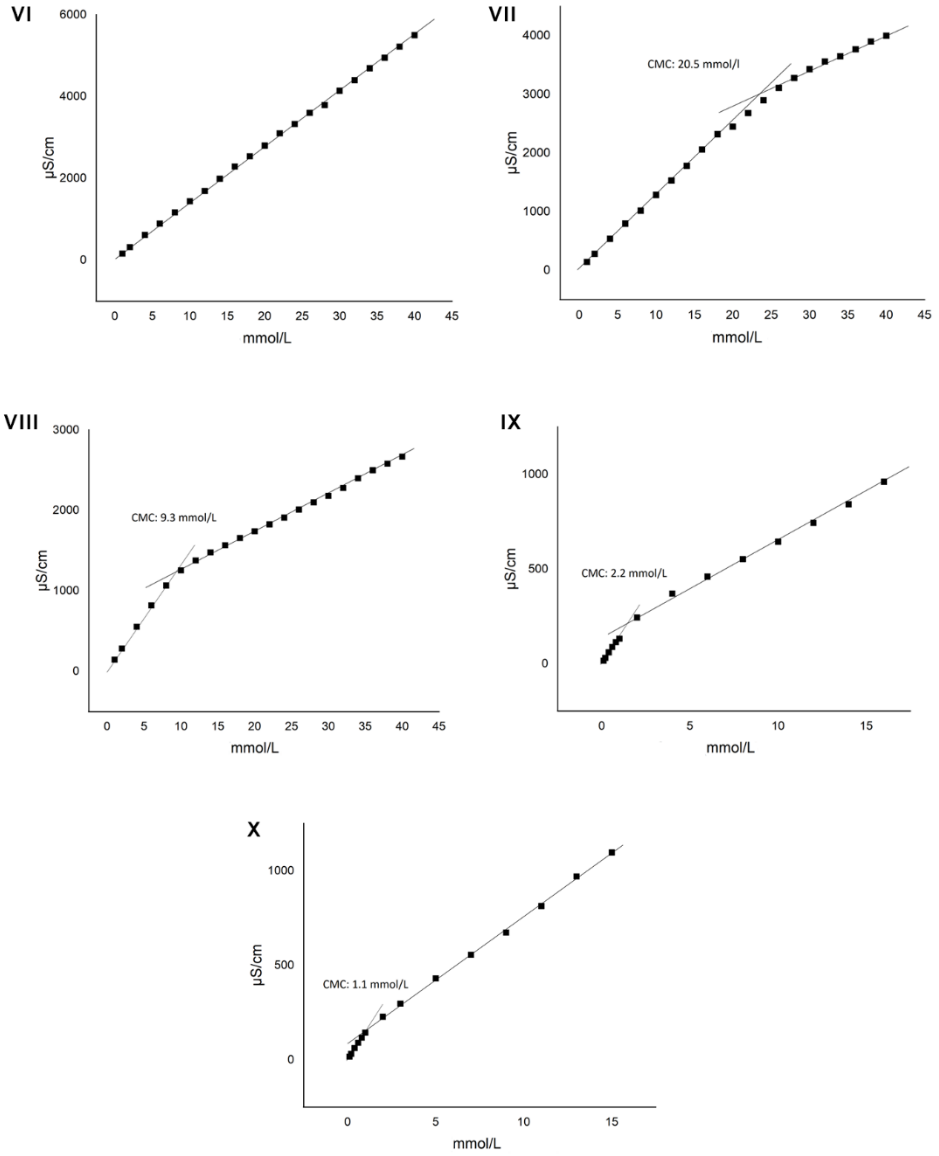

LogP calculation method

The logP values (octanol/water partition coefficients) were calculated in order to estimate the lipophilicity character of compounds. These calculations were achieved using MarvinSketch software (Marvin 16.8.22.0, 2019, ChemAxon (http://www.chemaxon.com).

V. ^{1}H AND ^{13}C NMR SPECTRA

(**I**) 4,6-dichloro-*N*-decyl-1,3,5-triazin-2-amine.

^{1}H NMR

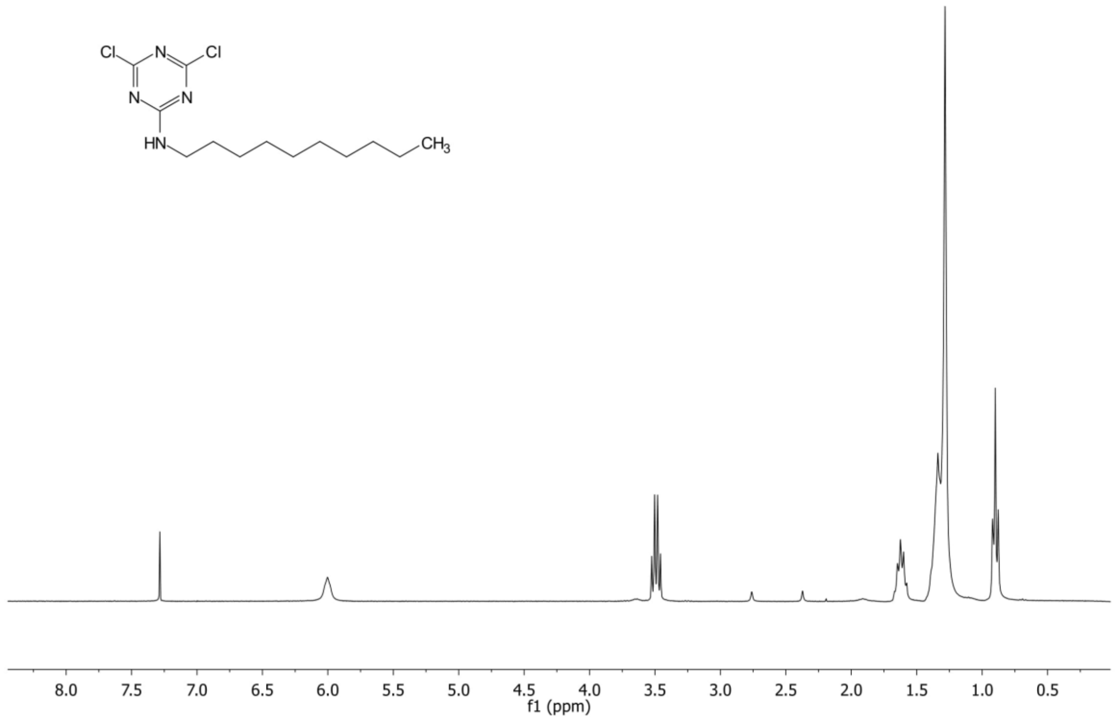

^{13}C NMR

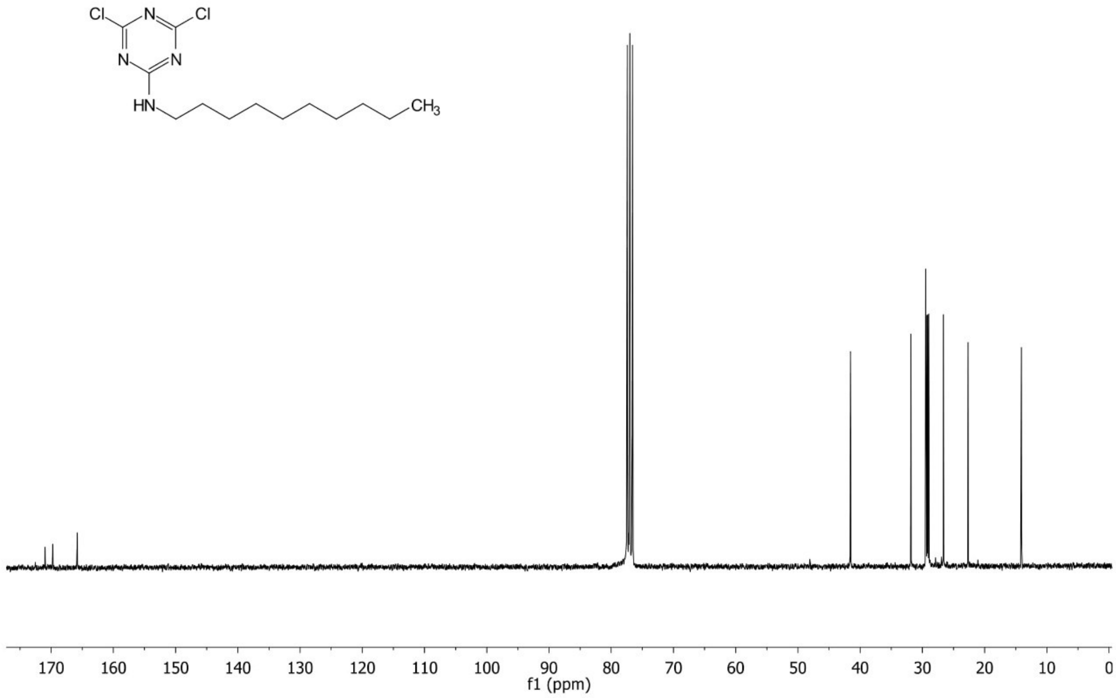

(**II**) 4,6-dichloro-*N*-dodecyl-1,3,5-triazin-2-amine.

^{1}H NMR

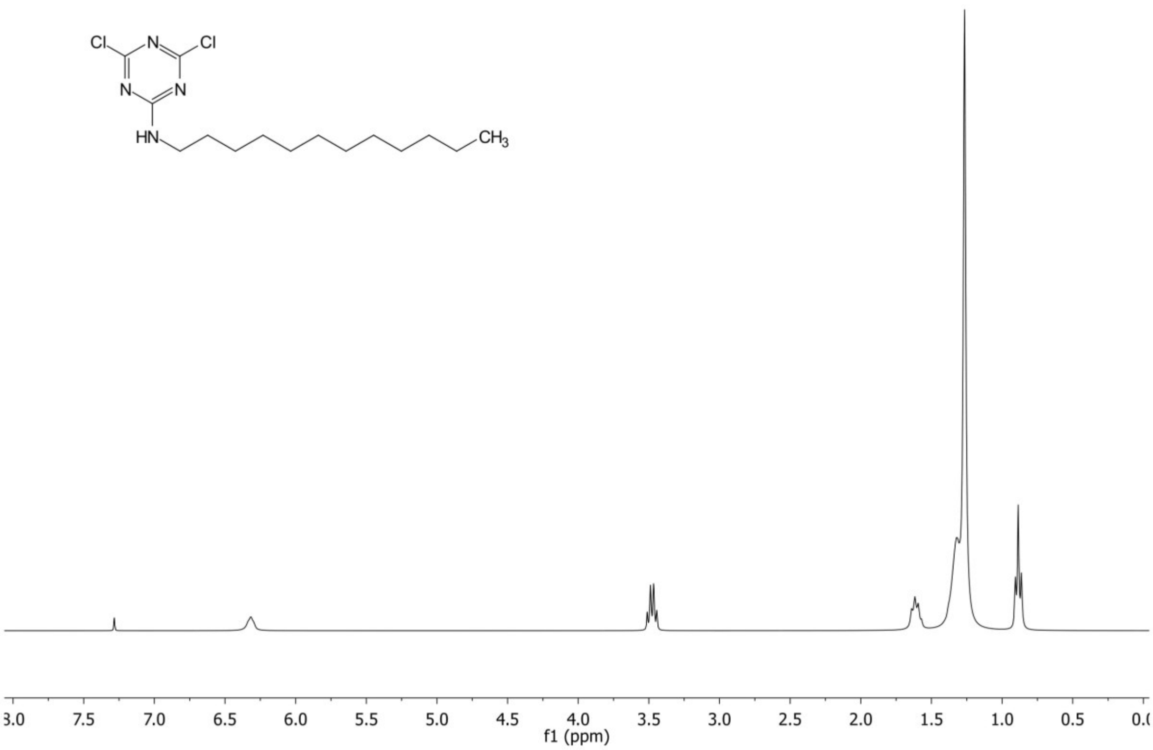

^{13}C NMR

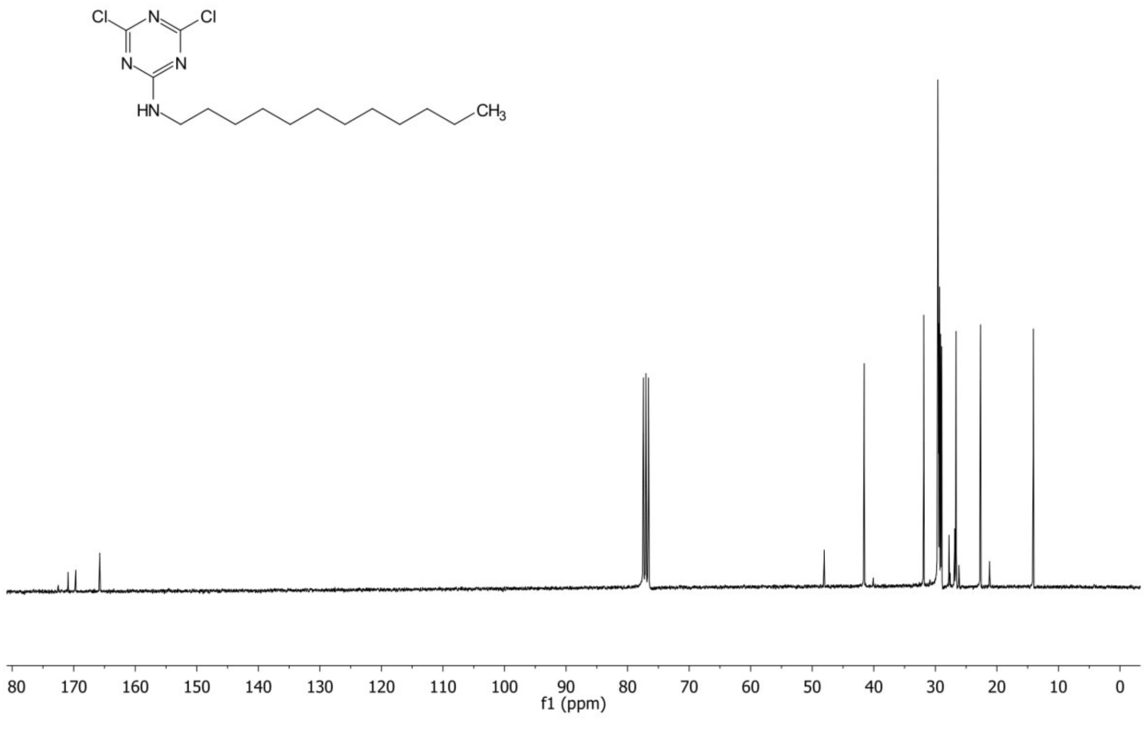

(**III**) 4,6-dichloro-*N*-tetradecyl-1,3,5-triazin-2-amine.

^{1}H NMR

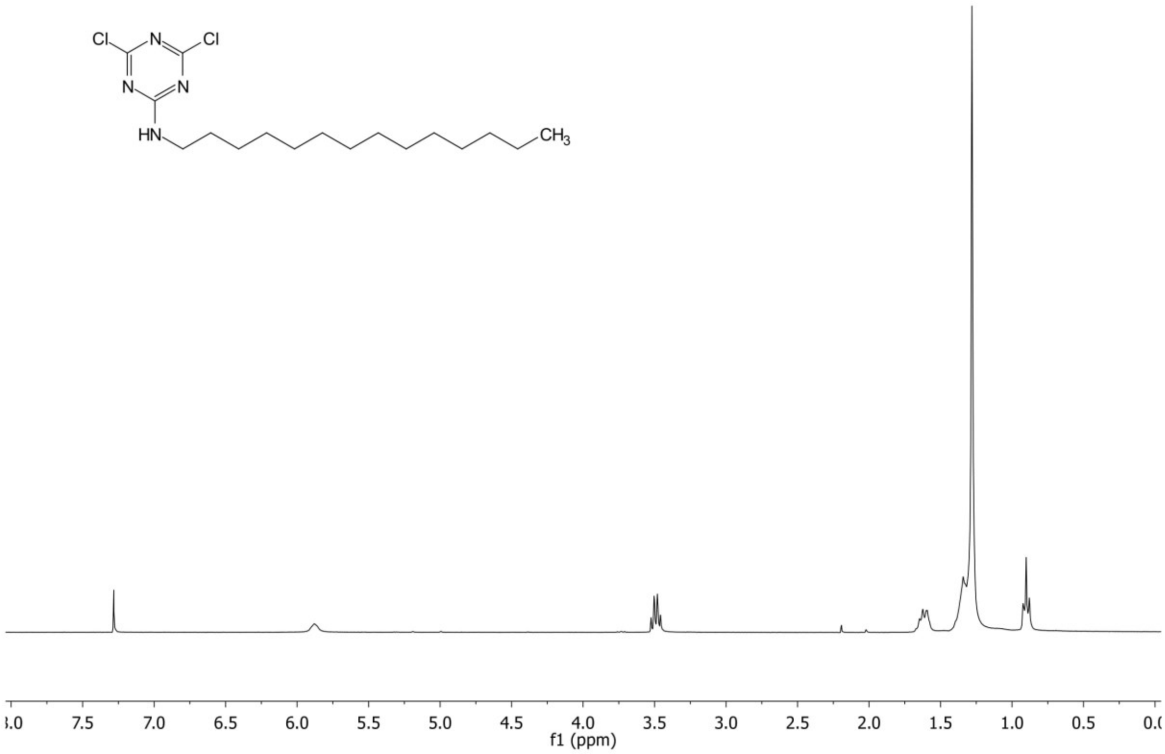

^{13}C NMR

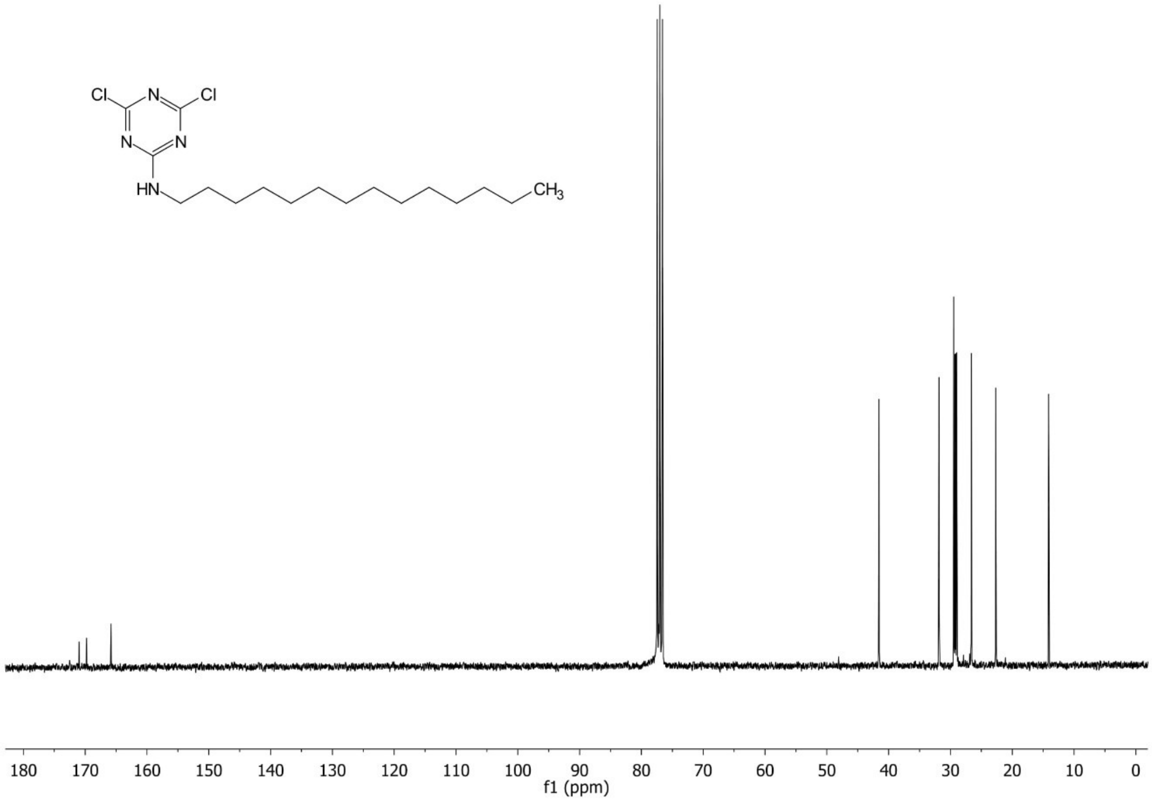

(**IV**) 4,6-dichloro-*N*-hexadecyl-1,3,5-triazin-2-amine.

^{1}H NMR

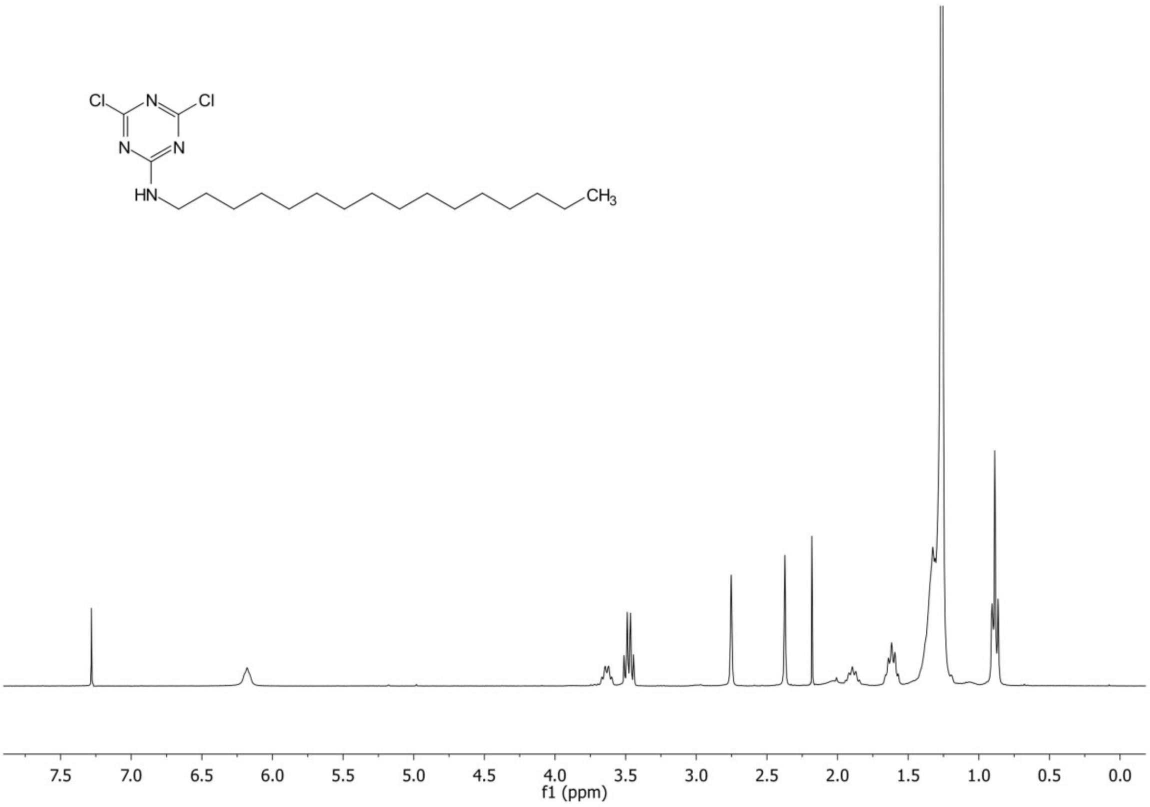

^{13}C NMR

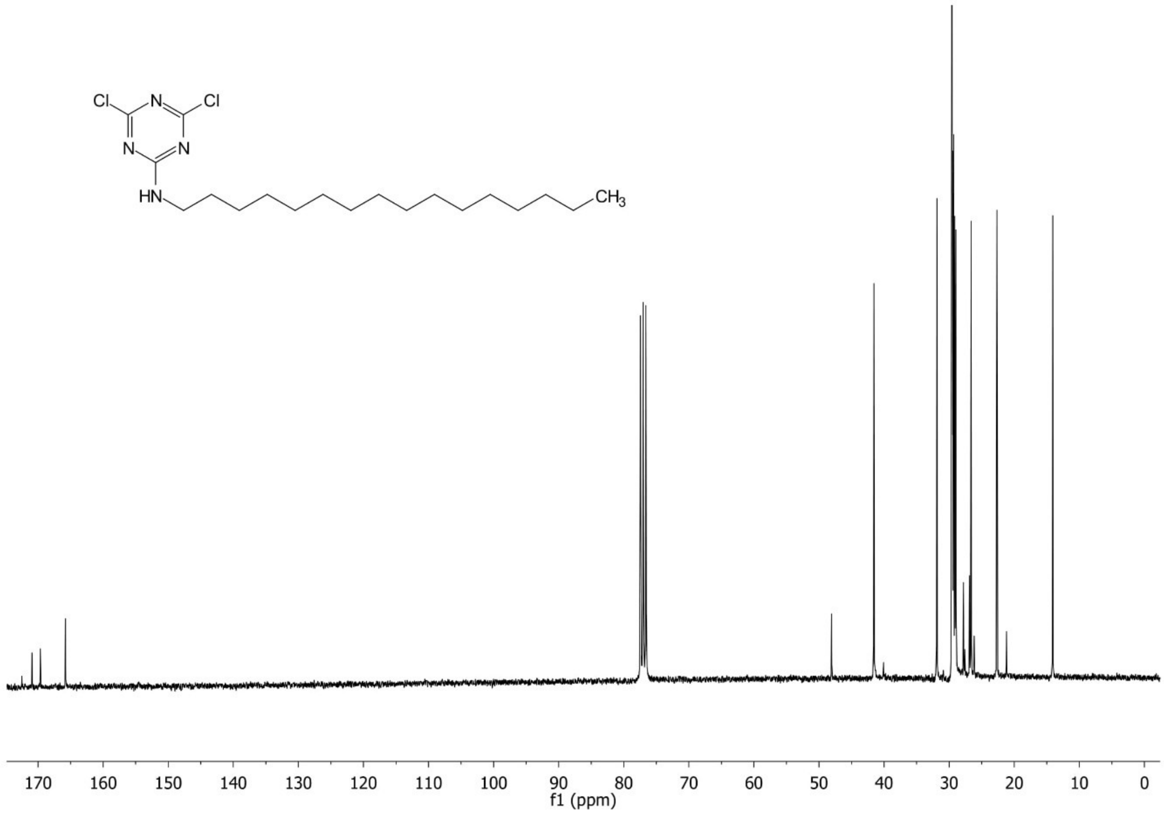

(**V**) 4,6-dichloro-*N*-octadecyl-1,3,5-triazin-2-amine.

^{1}H NMR

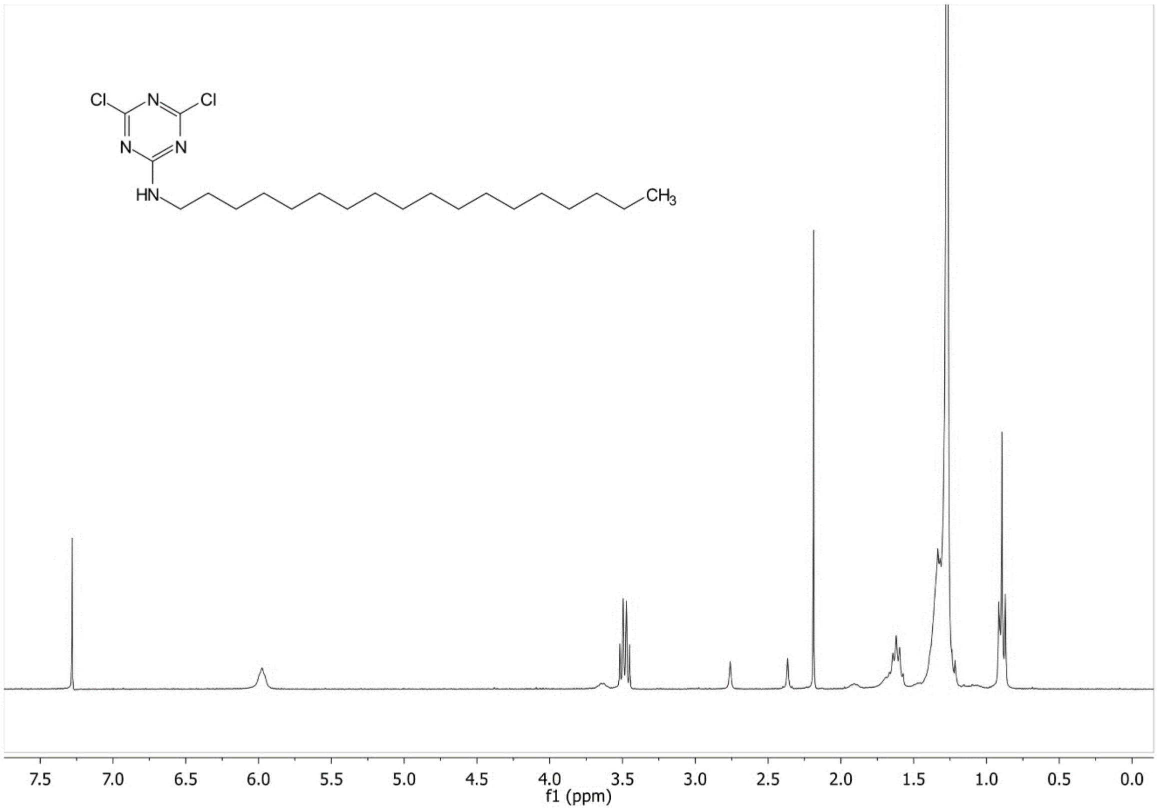

^{13}C NMR

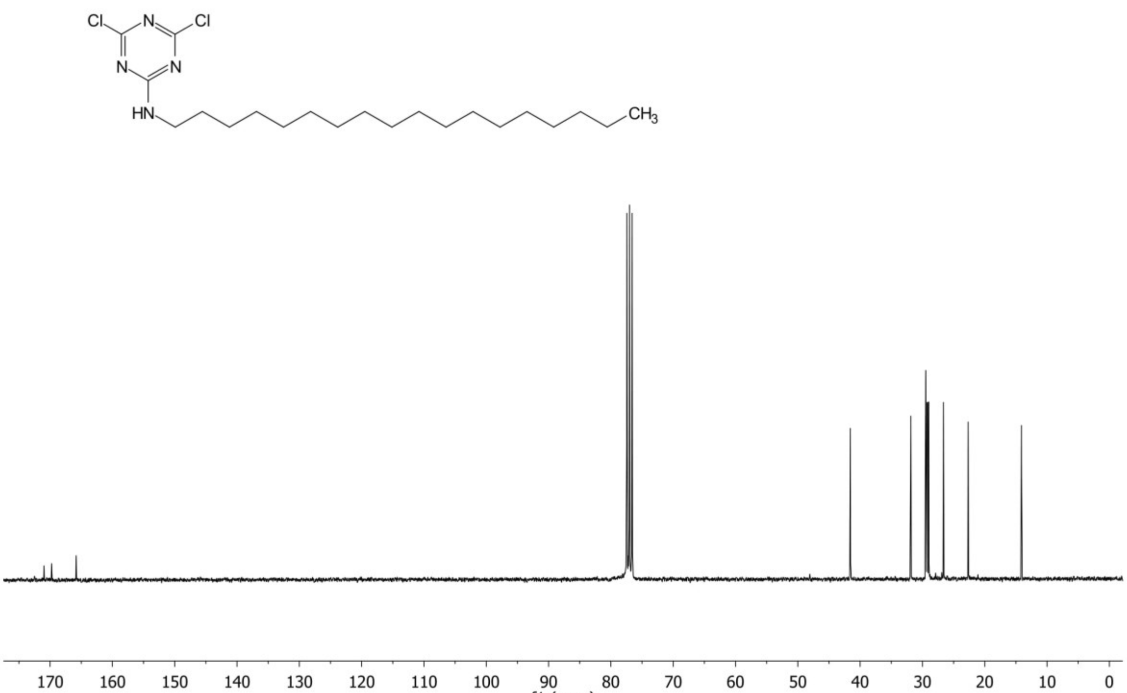

(**VI**) 3,3'-(6-(decylamino)-1,3,5-triazine-2,4-diyl)bis(1-methyl-1H-imidazol-3-ium) chloride.

^{1}H NMR

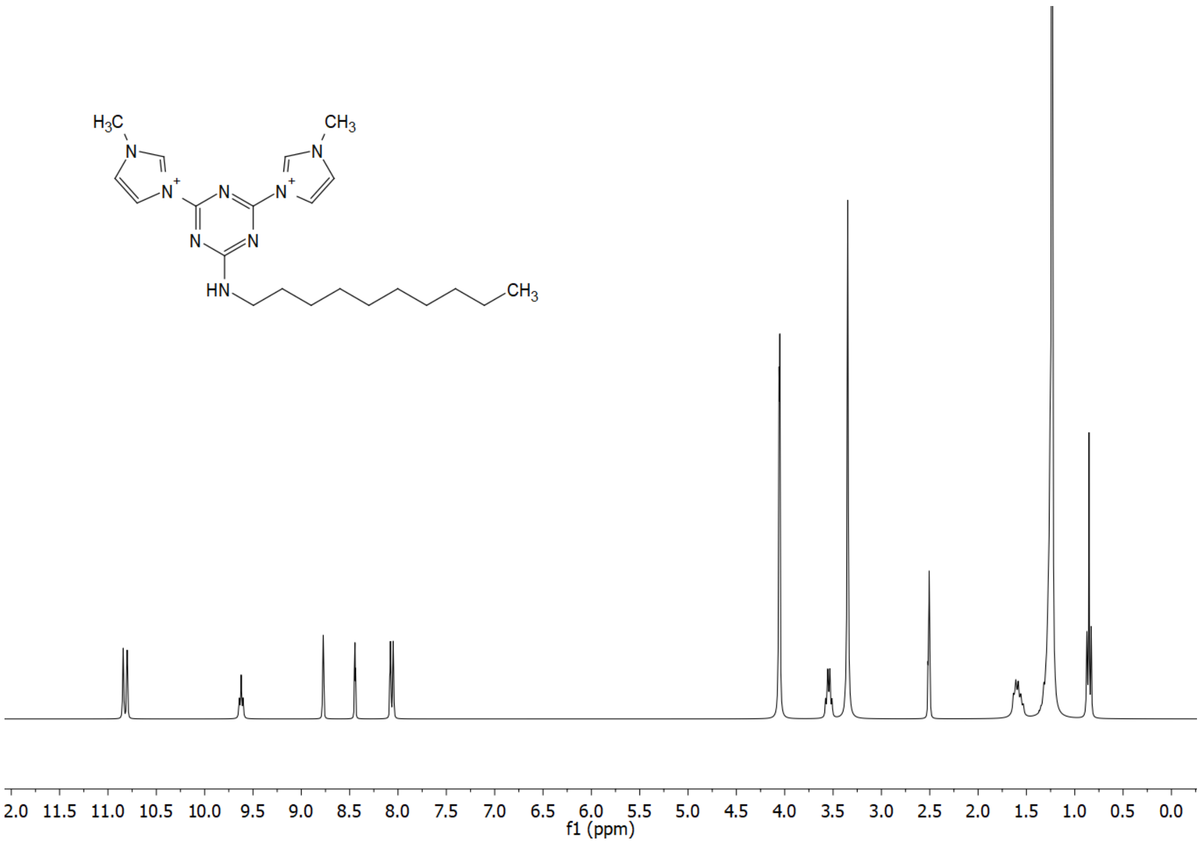

^{13}C NMR

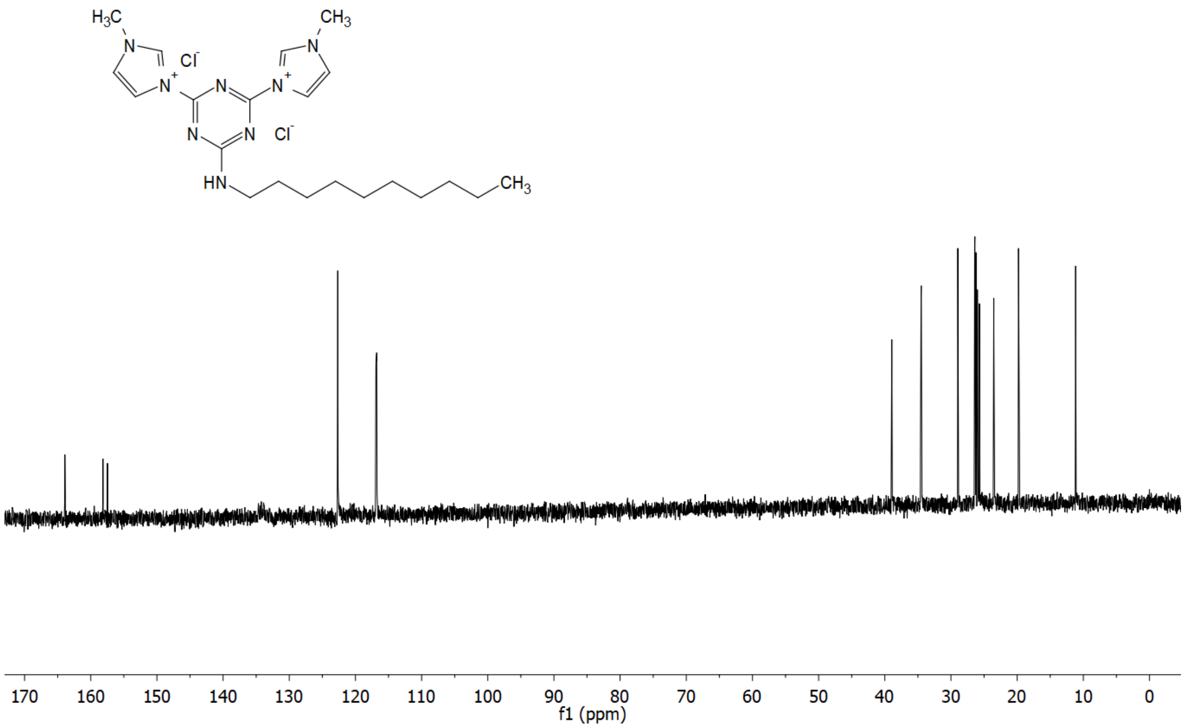

(**VII**) 3,3'-(6-(dodecylamino)-1,3,5-triazine-2,4-diyl)bis(1-methyl-1H-imidazol-3-ium) chloride**.**

^{1}H NMR

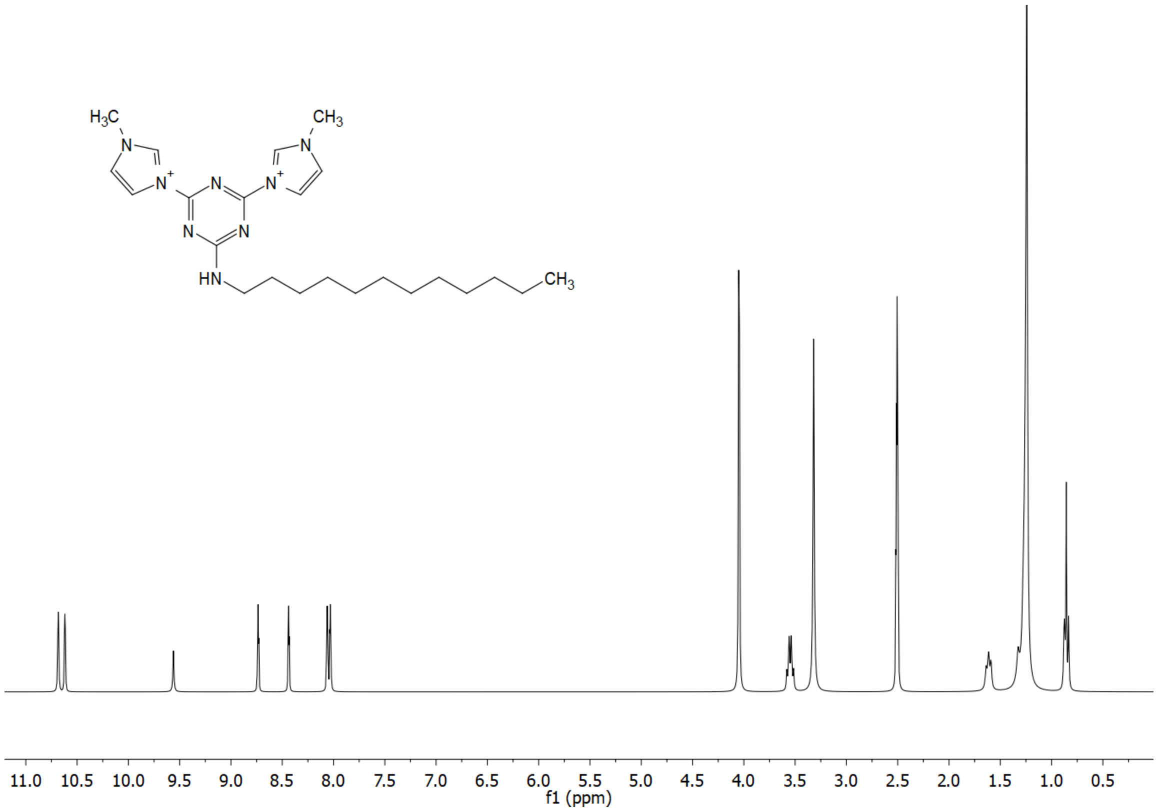

^{13}C NMR

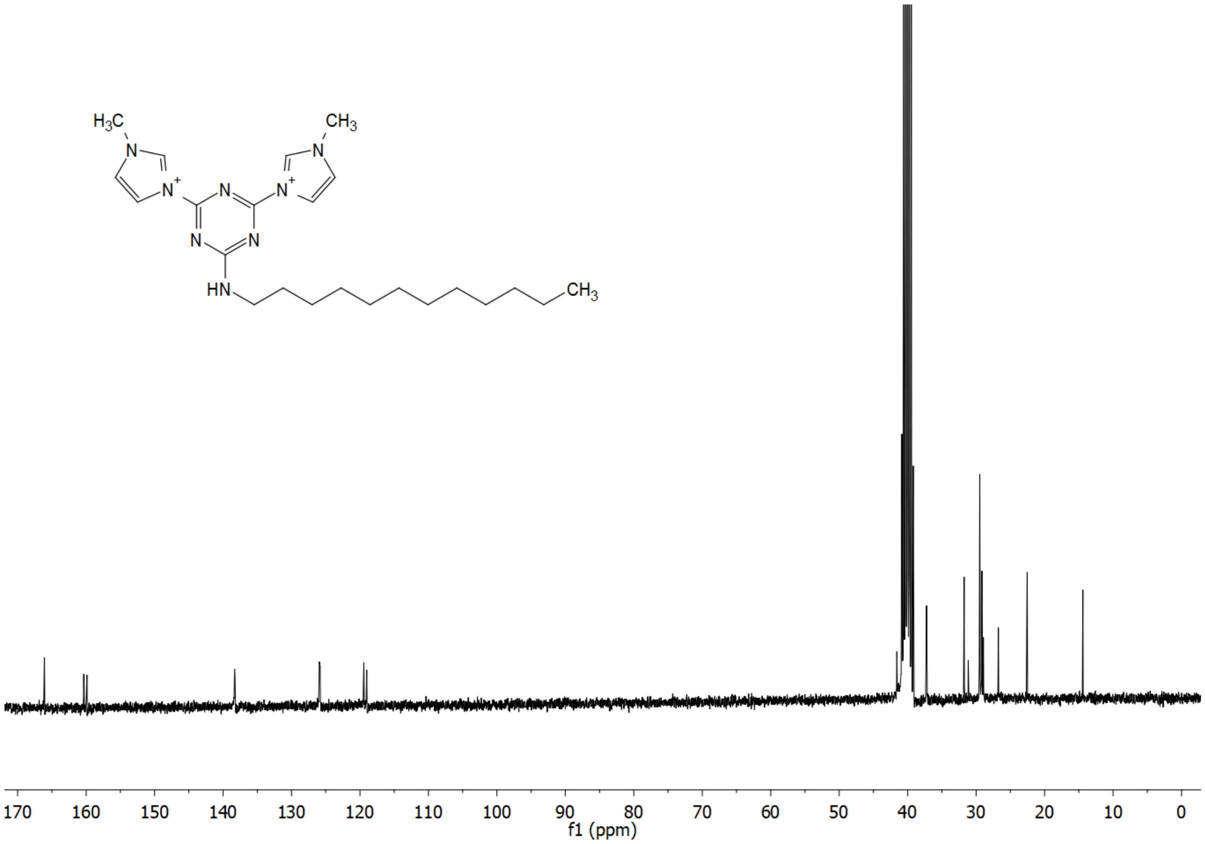

(**VIII**) 3,3'-(6-(tetradecylamino)-1,3,5-triazine-2,4-diyl)bis(1-methyl-1H-imidazol-3-ium) chloride.

^{1}H NMR

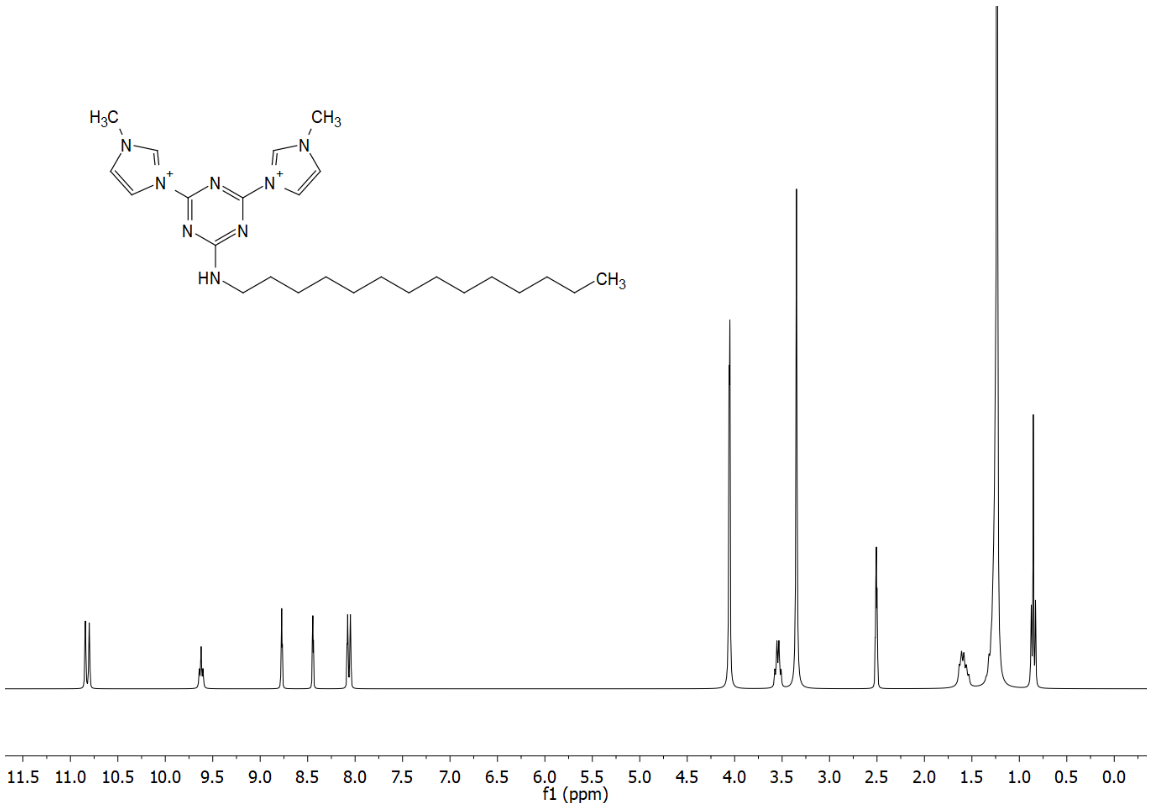

^{13}C NMR

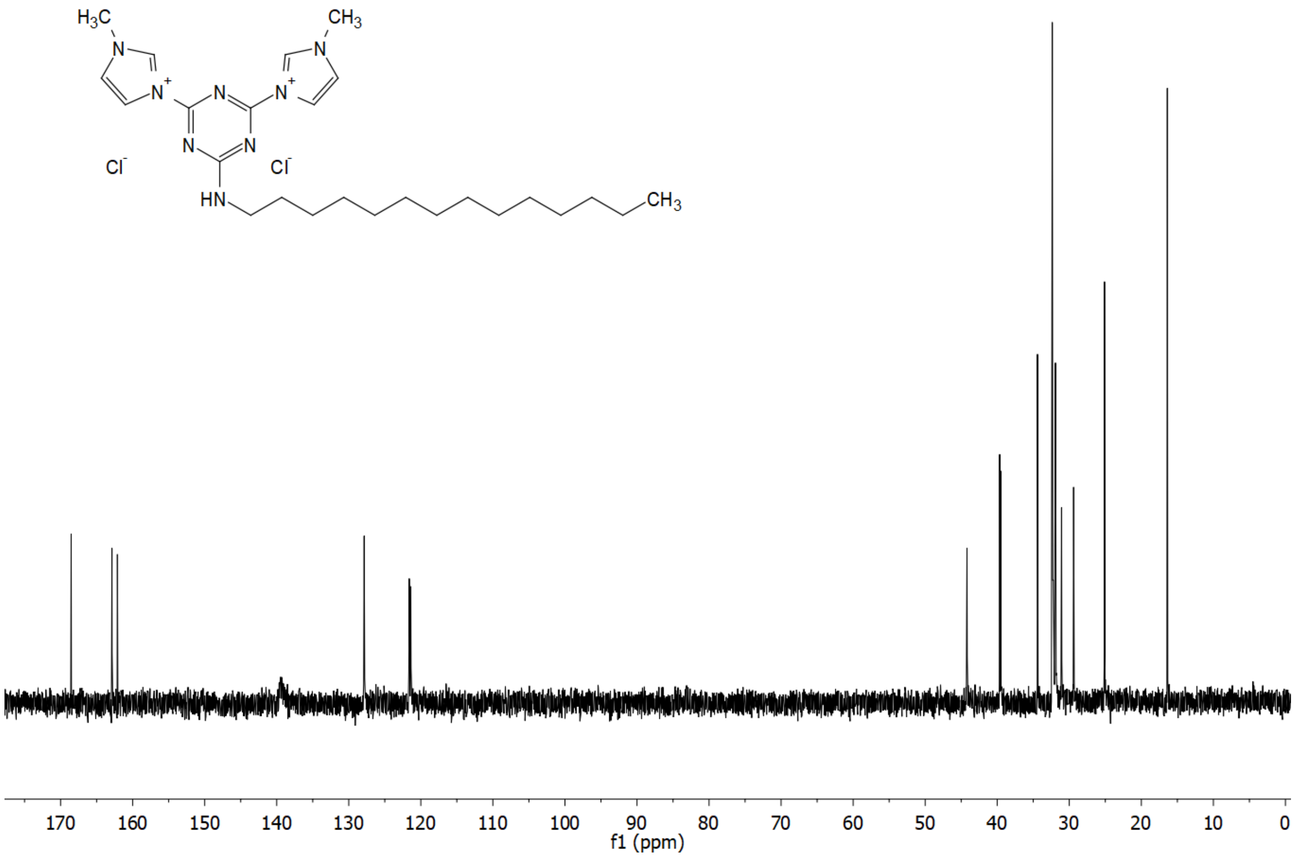

(**IV**) 3,3'-(6-(hexadecylamino)-1,3,5-triazine-2,4-diyl)bis(1-methyl-1H-imidazol-3-ium) chloride.

^{1}H NMR

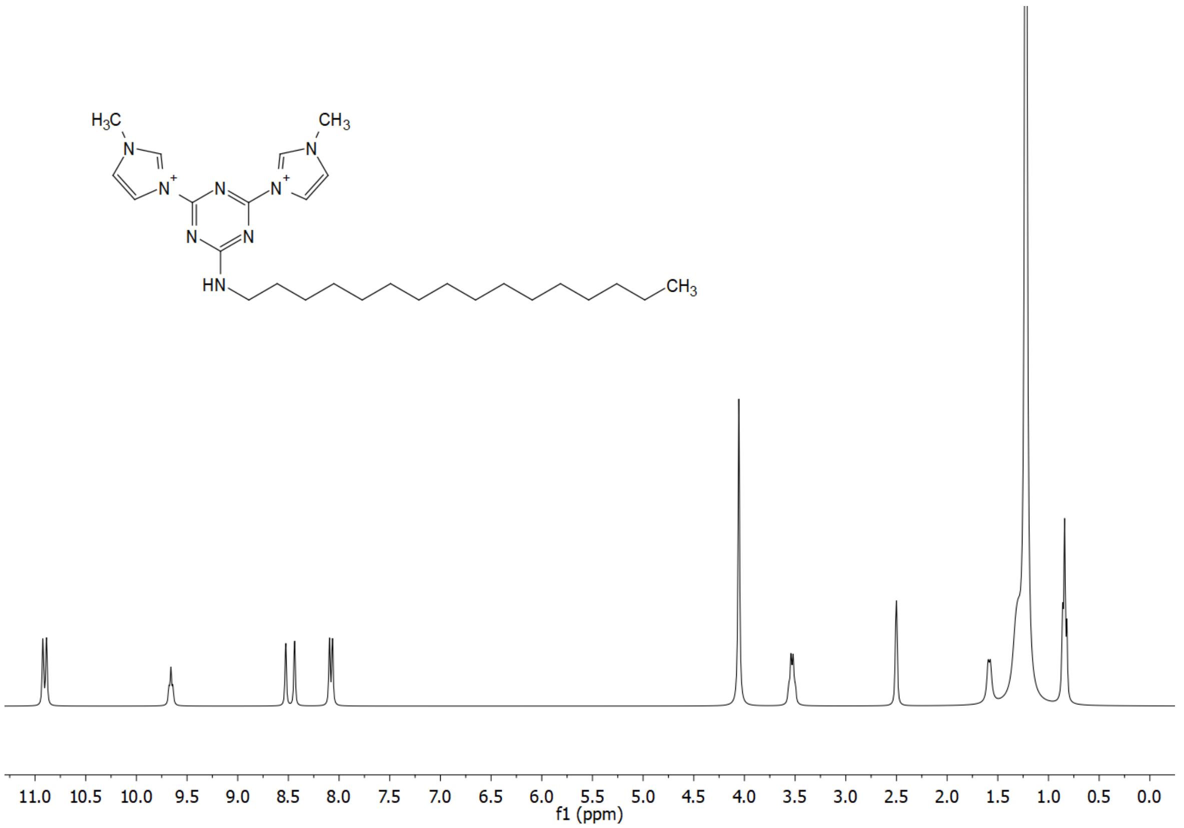

^{13}C NMR

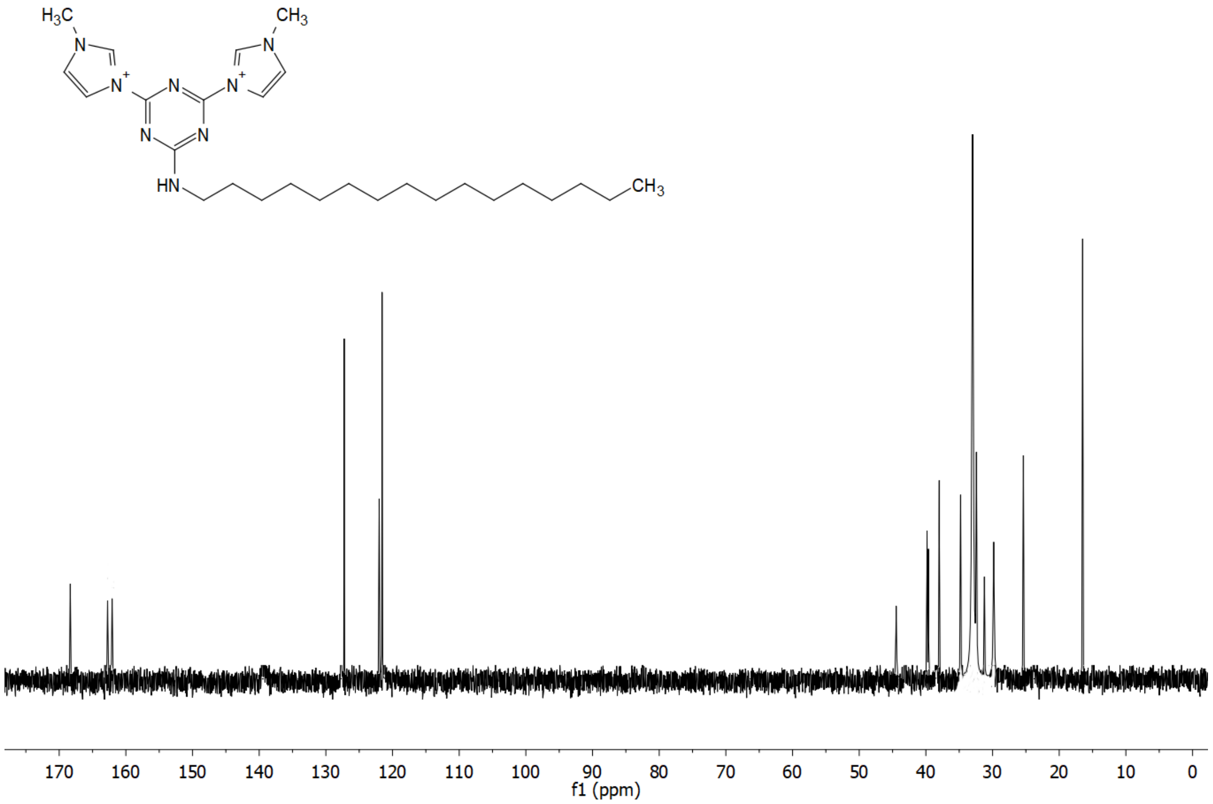

(**X**) 3,3'-(6-(octadecylamino)-1,3,5-triazine-2,4-diyl)bis(1-methyl-1H-imidazol-3-ium) chloride.

^{1}H NMR

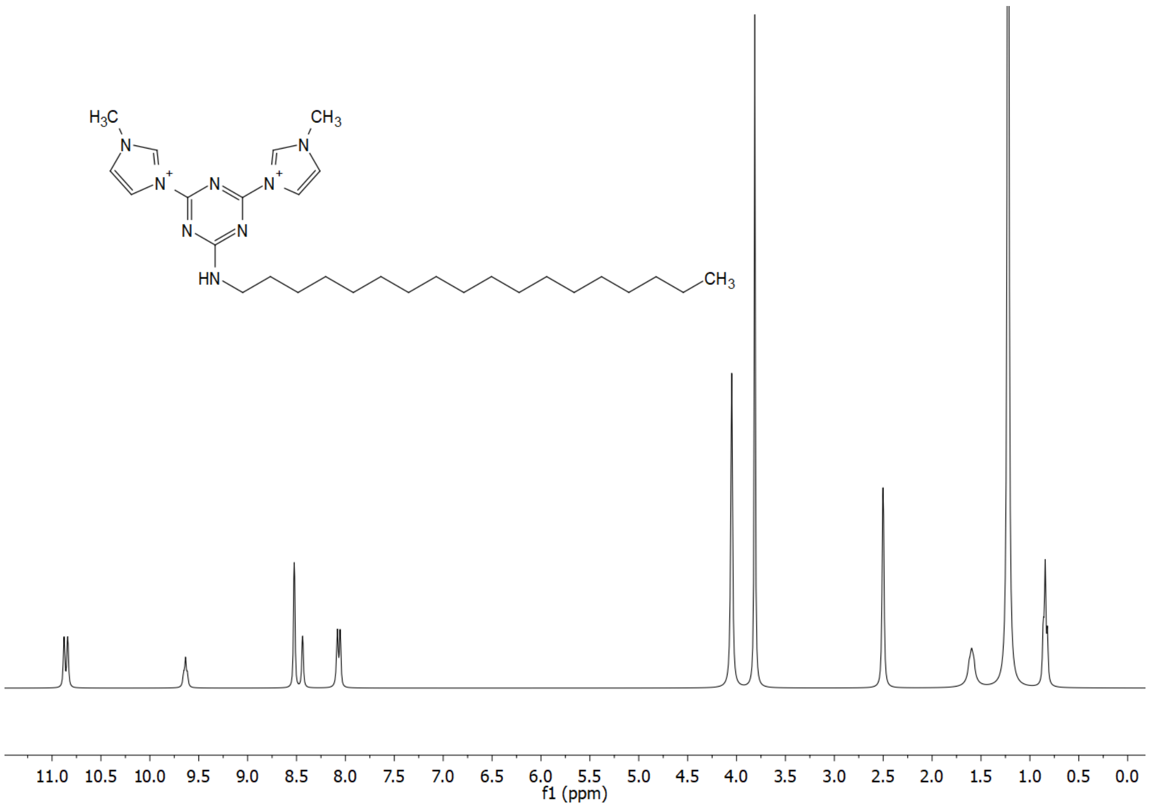

^{13}C NMR

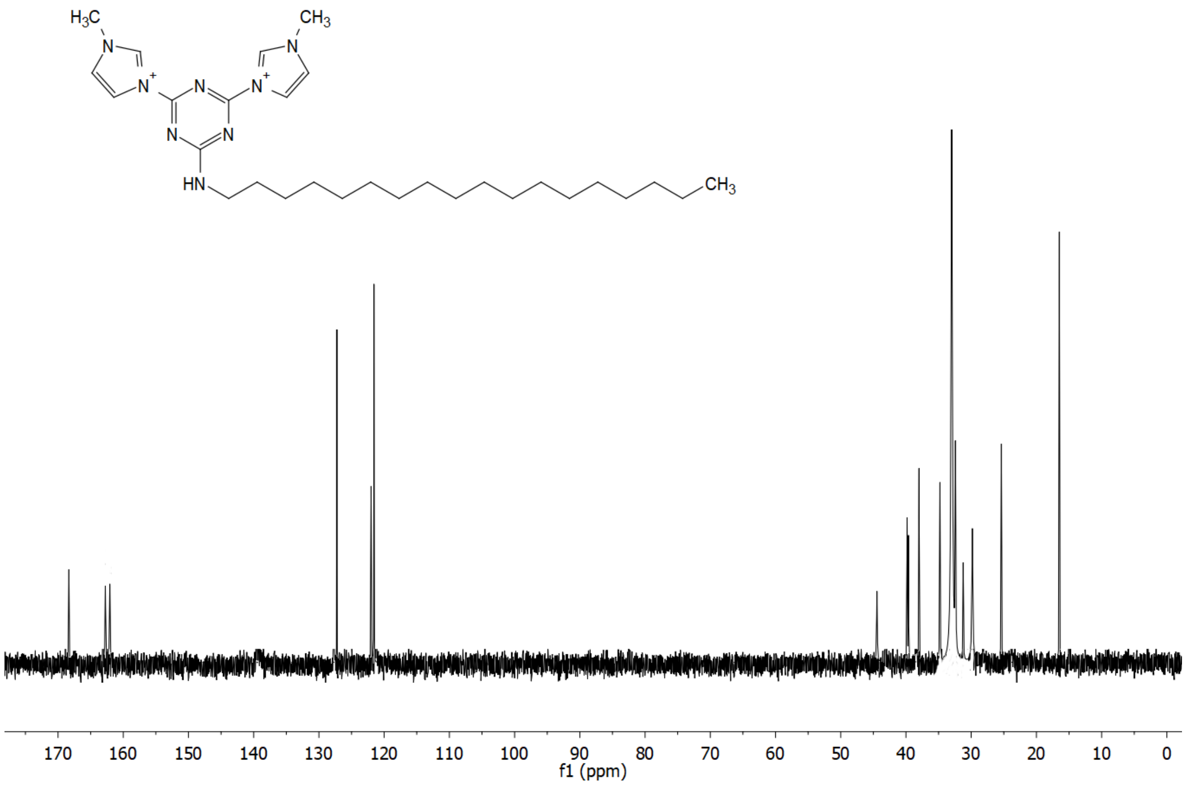

Supplement: RA-011-D1RA03455C-s001 [file RA-011-D1RA03455C-s001.pdf]
